# Wild-type IDH2 is a therapeutic target for triple-negative breast cancer

**DOI:** 10.1038/s41467-024-47536-6

**Published:** 2024-04-24

**Authors:** Jiang-jiang Li, Tiantian Yu, Peiting Zeng, Jingyu Tian, Panpan Liu, Shuang Qiao, Shijun Wen, Yumin Hu, Qiao Liu, Wenhua Lu, Hui Zhang, Peng Huang

**Affiliations:** 1 State Key Laboratory of Oncology in South China, Collaborative Innovation Center for Cancer Medicine, Sun Yat-sen University Cancer Center, 651 Dongfeng East Road, Guangzhou, 510060 China; 2 Metabolic Innovation Center, Sun Yat-sen University Zhongshan School of Medicine, Guangzhou, 510080 China; 3 Department of Medical Oncology, Sun Yat-sen University Cancer Center, 651 Dongfeng East Road, Guangzhou, 510060 China

**Keywords:** Breast cancer, Molecular medicine, Cancer metabolism

## Abstract

Mutations in isocitrate dehydrogenases (IDH) are oncogenic events due to the generation of oncogenic metabolite 2-hydroxyglutarate. However, the role of wild-type IDH in cancer development remains elusive. Here we show that wild-type IDH2 is highly expressed in triple negative breast cancer (TNBC) cells and promotes their proliferation in vitro and tumor growth in vivo. Genetic silencing or pharmacological inhibition of wt-IDH2 causes a significant increase in α-ketoglutarate (α-KG), indicating a suppression of reductive tricarboxylic acid (TCA) cycle. The aberrant accumulation of α-KG due to IDH2 abrogation inhibits mitochondrial ATP synthesis and promotes HIF-1α degradation, leading to suppression of glycolysis. Such metabolic double-hit results in ATP depletion and suppression of tumor growth, and renders TNBC cells more sensitive to doxorubicin treatment. Our study reveals a metabolic property of TNBC cells with active utilization of glutamine via reductive TCA metabolism, and suggests that wild-type IDH2 plays an important role in this metabolic process and could be a potential therapeutic target for TNBC.

## Abstract (web summary)

Isocitrate dehydrogenase (IDH) mutations are associated with cancer development and IDH-mutant inhibitors are approved to treat IDH-mutant cancer. Here, the authors show in preclinical murine models that wild-type IDH2 is a potential therapeutic target for triple-negative breast cancer.

## Introduction

Metabolic alterations are consistently observed in various types of cancer cells, and have been considered as a major hallmark of cancer^1^. The increase in aerobic glycolysis, known as the Warburg effect, represents a most well-studied metabolic phenomenon in cancer cells and constitutes a biochemical basis of the ^18^F-FDG PET-CT scan for cancer diagnosis due to the elevated uptake of glucose by cancer tissues^2^. The discovery that mutations in isocitrate dehydrogenases (IDH1 or IDH2) could drive cancer development due to the abnormal generation of an oncometabolite 2-hydroxyglutarate is another example of the direct involvement of altered metabolism in carcinogenesis. In fact, IDH1 and IDH2 mutations have been detected in a majority of low-grade gliomas and secondary high-grade gliomas^3^, and in approximately 20% of acute myeloid leukemia (AML)^4^. Mechanistically, IDH mutations lead to cancer development due in part to certain changes in epigenetics and redox signaling induced by 2-hydroxyglutarate accumulation^5–7^. The important role of IDH mutations in cancer is further underscored by the fact that IDH inhibitors such as Ivosidenib and Enasidenib have been developed for cancer treatment^8^.

Despite the long-standing observations of cancer metabolic reprogramming and its successful applications to cancer diagnosis, attempts to use metabolic intervention strategies for cancer treatment have so far been met with mixed results^9^. Although inhibition of glycolysis seems to be a logical approach to abrogating cancer metabolism and thus might selectively impact the malignant cells, so far, no clinically effective glycolytic inhibitors have been successfully developed for the treatment of cancer patients^10^. This is due in part to the high metabolic flexibility of cancer cells, which are able to change their metabolic pathways in response to metabolic intervention^11,12^. For example, inhibition of glycolysis may induce a compensatory increase in mitochondrial OXPHOS in conjunction with the active utilization of glutamine and fatty acids as alternative fuels, rendering glycolytic inhibition ineffective. On the bright side, inhibitors of mutant IDH1 (Ivosidenib) and mutant IDH2 (Enasidenib) seem effective in certain leukemia patients, and have been approved by the FDA for the treatment of AML^8^. It is unclear, however, why these IDH inhibitors are effective only in certain AML but not in other cancers with IDH mutations. A possible explanation would be that besides IDH mutations, different cancers may intrinsically have different metabolic patterns, and thus their dependency on mutant IDHs may vary significantly. As such, elucidation of specific metabolic alterations in specific cancer types is highly important for the design of effective metabolic intervention strategies for cancer treatment.

IDH2 is located in the mitochondria and plays a key role in the Krebs cycle (also known as tricarboxylic acid cycle or TCA cycle) by catalyzing the reversible conversion between isocitrate and alpha-ketoglutarate (α-KG)^13^. Under normal physiological conditions, the metabolic flow from isocitrate to α-KG with the conversion of NADP^+^ to NADPH and a release of CO_2_ is the step in the oxidative TCA cycle catalyzed by IDH2. Reversely, the metabolic flow from α-KG to isocitrate represents the biochemical reaction of the reverse TCA cycle (also known as reductive TCA cycle), which is used in some bacteria to synthesize carbon compounds from CO_2_^14^. Although the reductive TCA cycle could occur in mammalian cells, its relative activity (flow rate) and biological significance remain to be defined^15^. Since α-KG is located at the cross point between the TCA cycle and the glutamine metabolic pathway, it is possible that the reductive conversion of α-KG to isocitrate may play a significant role in channeling glutamate into TCA cycle for anaplerotic utilization in biosynthesis of lipids via isocitrate/citrate, which might be important for cancer cells with active anabolism, especially in cells under hypoxia or with defective mitochondria^16–18^. Thus, it would be highly important to test this possibility and to evaluate the role of IDH2 in this metabolic process.

Although the impact of IDH2 mutation on cancer development through the generation of oncometabolite 2-hydroxyglutarate has been well-characterized, the role of wild-type IDH2 in cancer remains to be further defined. Our recent study showed that wild-type IDH2 was highly expressed in lung cancer cells and promoted tumor growth^19^. High expression of wild-type IDH2 was shown to contribute to the survival of lung cancer cells, esophageal squamous cell carcinoma, and hepatocellular carcinoma cells^19–21^. Microarray and proteomic analysis identified high levels of IDH2 to be a factor of poor overall survival in breast cancer, but the underlying mechanisms remain elusive^22,23^. In this study, we use genetic, biochemical, and pharmacological approaches to investigate the role of wild-type IDH2 in breast cancer, and found that this enzyme plays a critical role in the survival of triple-negative breast cancer (TNBC) cells by promoting the reductive TCA cycle and keeping α-KG at the normal range. Abrogation of wild-type IDH2 led to aberrant accumulation of α-KG, which in turn exerted a double hit on energy metabolism leading to ATP depletion and TNBC cell death. Our study revealed an active reductive TCA cycle in TNBC cells that has not been appreciated previously, and suggested that IDH2 could be an effective therapeutic target in TNBC.

## Results

### High expression of wild-type IDH2 in TNBC is essential to support tumor growth

**Fig. 1:**
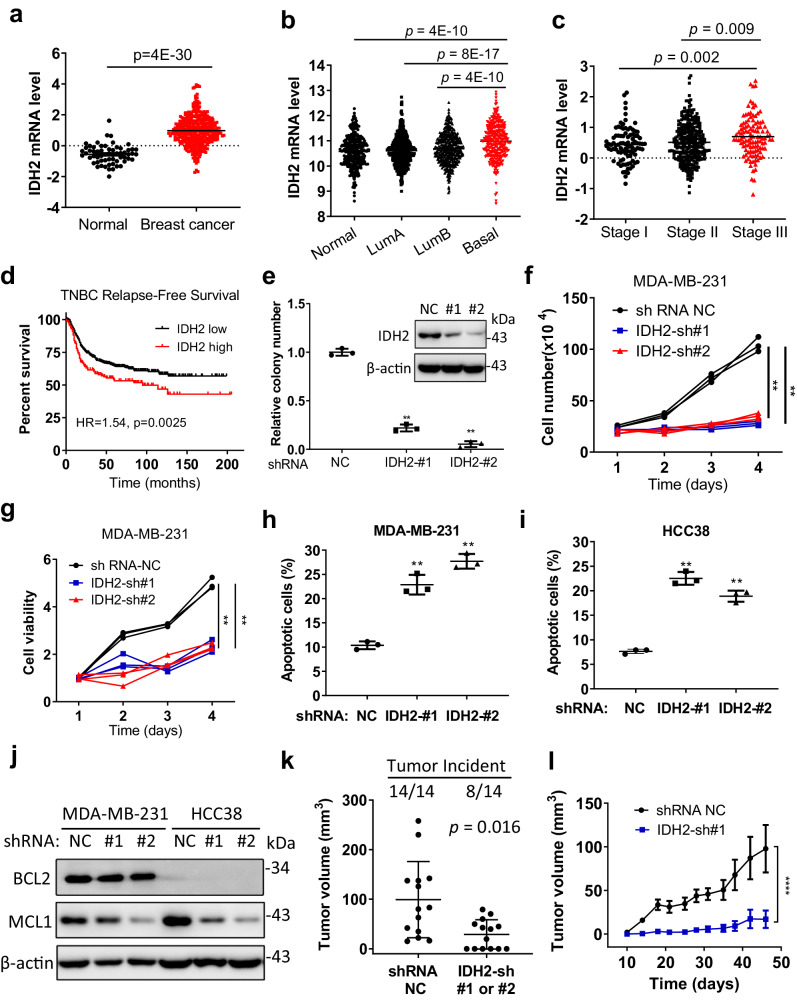
High expression of wild-type IDH2 in TNBC is essential to support tumor growth. **a**–**c** Analysis of IDH2 expression in **a** breast cancer tissues (*n* = 388) and normal tissues (*n* = 61, Oncomine breast cancer datasets dataset), **b** across different subtypes of breast cancer tissues (normal *n* = 333, LumA *n* = 511, LumB *n* = 416, basal *n* = 315, Survexpress breast cancer datasets), or **c** different disease stages (Stage I *n* = 92, stage II *n* = 299, stage III *n* = 112, Survexpress breast cancer datasets). **d** Censored Cox analysis of Kaplan–Meier survival curves of TNBC patients (*n* = 316) stratified by IDH2 high- and low-expression groups, which were analyzed for relapse-free survival using the median value of IDH2 expression as the cut-off (Kmplot). **e**–**h** Colony number (**e**), cell number (**f**), cell viability (**g**), and apoptosis ratio (**h**) of MDA-MB-231 cell stably transfected with IDH2 shRNA (#1 and #2) or non-targeting control shRNA (NC). The samples for western blot analysis of IDH2 and β-actin were derived from the same experiment and analyzed in parallel in separate gels. Colonies were counted on day 14 (*n* = 3 for each condition, technical replicates, representative of two independent experiments (original data included in the Source Data). **i** Apoptosis in HCC38 cells transfected with IDH2 shRNA or control shRNA. Cell death was quantified using Annexin V-FITC/PI staining followed by flow cytometry analysis, *n* = 3 for each condition (technical replicates, representative of two independent experiments, original data included in the Source Data). **j** Western blot analysis of key survival proteins (BCL2 and MCL-1) in MDA-MB-231 or HCC38 cells transfected with IDH2 shRNA (#1 and #2) or non-targeting control shRNA (NC). The samples derived from the same experiment but different gels (one gel for BCL2, another for MCL-1, and another for β-actin) were processed in parallel. Images are representative of two independent experiments (original data included in Source Data)；**k** Tumor incident and tumor size of Balb/C-nude mice implanted with MDA-MB-231 cells without or with IDH2 knockdown. The difference between the NC control group and the IDH2 knockdown groups (sh#1 or sh#2) was analyzed using the Chi-square test (SPSS version 17, *n* = 14 for each group, independent biological repeats). **l** Tumor growth curves of xenografts in Balb/C-nude mice implanted with MDA-MB-231 cells stably transfected with IDH2 shRNA (#1) or control shRNA (NC), *n* = 7 for each group, independent biological repeats; two-way ANOVA test was employed for statistical analysis, ****p ≤ 0.0001. Error bars in all panels represent mean ± SD. A two-sided Student’s *t*-test was employed for statistical analysis. *p ≤ 0.05; **p ≤ 0.01; ***p ≤ 0.001.

Although IDH2 mutations are well-known oncogenic events in several cancer types, the role of wild-type IDH2 in cancer remains elusive. Our analysis of the Cancer Genome Atlas (TCGA) datasets and Gene Expression Omnibus (GEO) revealed that wild-type IDH2 mRNA expression was significantly elevated in breast cancer compared with normal tissues (Fig. 1a and Supplementary Table S1). The increased IDH2 mRNA in breast cancer was most notable in the basal type (Fig. 1b) and appeared positively correlated with clinical stages (Fig. 1c). The expression of IDH2 protein in normal and breast cancer tissues (including TNBC and non-TNBC tumor samples) was analyzed using immunohistochemistry staining. The results showed that both TNBC and non-TNBC tumor tissues expressed significantly higher levels of IDH2 protein compared to the normal breast tissues, with the highest level observed in TNBC tumor tissues (Supplementary Fig. S1a, b). Importantly, patient outcome analysis showed that higher IDH2 expression was correlated with worse clinical outcomes of the TNBC patients, as evidenced by a significantly shorter relapse-free survival time in the IDH2-high expression group (Fig. 1d). The increase in IDH2 mRNA expression in breast cancer seemed notably associated with genomic DNA amplification (Supplementary Fig. S1c) and the gain of copy number in TNBC (Supplementary Fig. S1d). Higher IDH2 expression was also correlated with worse overall survival ((Supplementary Fig. S1e) and post-progression survival (Supplementary Fig. S1f). These data together suggest that high expression of wild-type IDH2 might be important in promoting TNBC cancer progression.

Multiple approaches were then employed to evaluate the role of wild-type IDH2 expression in TNBC cell proliferation in vitro and tumor growth in vivo. First, a shRNA technique was used to knockdown the expression of IDH2 in TNBC cells (MDA-MB-231) with wild-type IDH2. As shown in Fig. 1e, IDH2-shRNA sequences #1 and #2 were effective in suppressing the target gene expression, which led to a significant inhibition of MDA-MB-231 cells to form colonies in culture. Consistently, direct cell counting and MTS assay also showed that the knockdown of IDH2 severely suppressed TNBC cell proliferation (Fig. 1f, g). Similar results were obtained in two other TNBC cell lines, BT549 and HCC38 (Supplementary Fig. S2a–c). To test the effect of IDH2 KD in non-TNBC cell lines, we used shRNA to suppress IDH2 expression in MCF7 and BT474 cells, and showed that such knockdown also substantially inhibited cell proliferation (Supplementary Fig. S2d, e). We also created gain-of-function models by stable overexpression of IDH2 in TNBC cells using lentiviral vectors, and showed that IDH2 overexpression could enhance cell proliferation in vitro (Supplementary Fig. S2f, g). The apparent lack of correlation between the levels of IDH2 KD and the extent of cytotoxicity was likely due to a compensatory increase in IDH1 and IDH3 expression in sh-IDH2 #2 cells. The expression of IDH1, IDH3α, β, and γ was found significantly elevated in sh-IDH2 #2 cells but not in sh-IDH2 #1 cells (Supplementary Fig. S2h). To further rule out the potential off-target effect, we performed a rescue experiment by constructing an IDH2 expression vector specifically resistant to IDH2 shRNA #2 and using it to re-express IDH2 in HCC38 and MDA-MD-231 cells pre-knocked down with IDH2 shRNA #2. The results showed that re-expression of IDH2 could largely restore cell proliferation in both cell lines (Supplementary Fig. S2i, j). We also performed a similar rescue experiment by constructing an IDH2 expression vector specifically resistant to shRNA #1, and used this vector to re-express IDH2 in MDA-MB-231 cells that had been knocked down with IDH2 shRNA #1. The results consistently showed that the re-expression of IDH2 could largely restore the cell proliferation in these cell lines (Supplementary Fig. S2k, l). These results indicated that the suppression of cell growth in IDH2 KD cells was not due to an off-target effect. Apoptosis analysis showed that silencing of IDH2 by shRNA resulted in a significant increase of annexin V-positive cells (Fig. 1h, i), accompanied by a reduced expression of survival molecule MCL-1 (Fig. 1j). Instant expression of shRNA#1 resistant IDH2 mRNA in stable IDH2 knockdown TNBC cells increased the MCL-1 protein level (Supplementary Fig. S2m), further confirming that wild-type IDH2 is critical for TNBC cell viability. In vivo experiments using TNBC xenograft models showed that knocking down of IDH2 severely suppressed tumor growth (Fig. 1k, l). These data together suggest that wt-IDH2 might play a major role in supporting TNBC cell viability and tumor growth in vivo.

### Suppression of IDH2 leads to α-ketoglutarate accumulation in TNBC cells due to a decrease in reductive TCA cycle

**Fig. 2:**
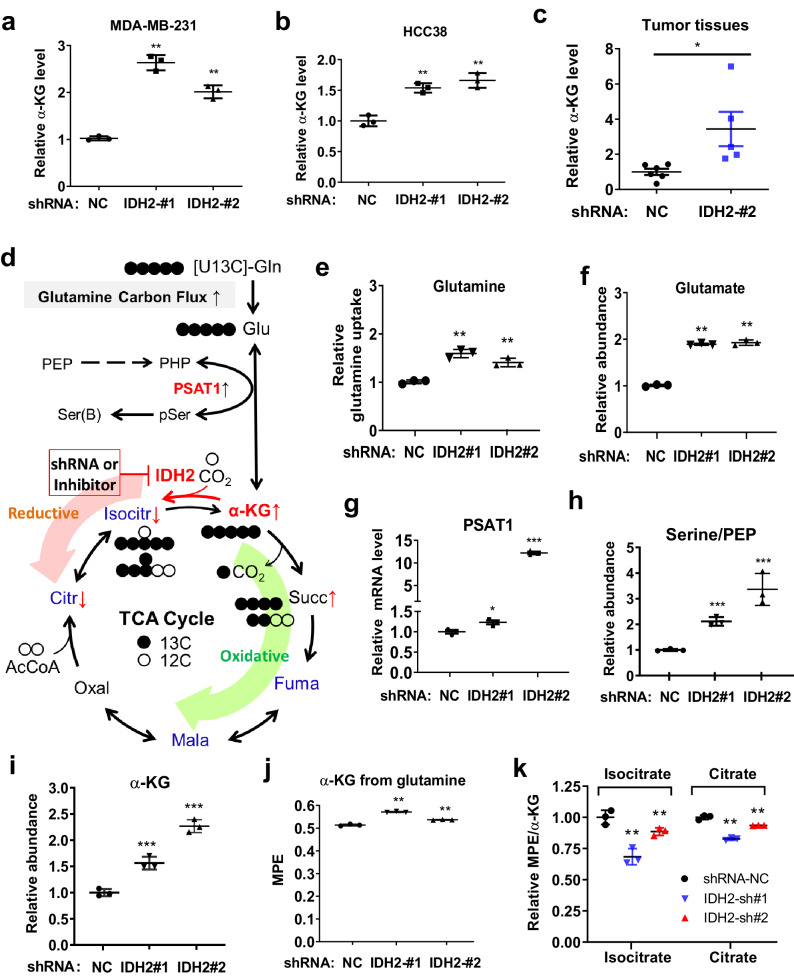
Suppression of IDH2 leads to accumulation of α-ketoglutarate in TNBC cells due to blockage of the reductive TCA cycle. **a** Effect of IDH2 knockdown by shRNA (IDH2-#1 and IDH2-#2) on intracellular α-ketoglutarate level in MDA-MB-231 cells. Non-target control shRNA (NC) was used as control. Cellular α-ketoglutarate was measured using an enzyme-based method as described in the method (*n* = 3, technical replicates, representative of two independent experiments, original data included in the Source Data). **b** Effect of IDH2 knockdown by shRNA (IDH2-#1 and IDH2-#2) on the cellular α-ketoglutarate level in HCC38 cells (*n* = 3, technical replicates, representative of two independent experiments, original data included in the Source Data file). **c** Relative α-ketoglutarate level in tumor tissues from mice inoculated with MDA-MB-231 cells transfected with IDH2 shRNA (*n* = 5, independent biological replicates) or non-target control RNA (NC, *n* = 6, independent biological replicates). **d** Schema of metabolic flux analysis of glutamine metabolic pathway feeding to the TCA cycle via α-ketoglutarate. The dark close circles represent [13C]-labeled carbons, whereas the open circles represent unlabeled 12C-carbons. The green arrow indicates the direction of oxidative TCA flow, while the orange arrow shows the direction of reductive TCA flow where IDH2 catalyzes the conversion of α-ketoglutarate to isocitrate. Inhibition of IDH2 led to an accumulation of α-ketoglutarate. **e**, **f** Comparison of glutamine uptake (**e**) and cellular glutamate (**f**) in MDA-MB-231 cells transfected with IDH2 shRNA (#1 and #2) or with non-target control RNA (NC). **g** Expression of PSAT1 in MDA-MB231 cells transfected with IDH2 shRNA (#1 and #2) or with non-target control RNA (NC). PSAT1 expression was measured by qRT-PCR. **h** Mole percent enrichment ratio of serine/PEP in MDA-MB231 cells transfected with IDH2 shRNA (#1 and #2) or with non-target control RNA (NC). **i**–**k** GC-MS analysis of α-KG abundance (**i**), its mole percent enrichment (MPE) (**j**), and the MPE/α-KG ratio in MDA-MB231 cells transfected with IDH2 shRNA (#1 and #2) or with non-target control RNA (NC) (**k**). Error bars in all panels represent mean values ± S.D., *n* = 3 (technical replicates) for each group in (**e**–**k**), a two-sided Student’s *t*-test was used to test the statistical significance. *p ≤ 0.05; **p ≤ 0.01; ***p ≤ 0.001.

Since IDH2 catalyzes the reaction between isocitrate and α-ketoglutarate (α-KG) in the TCA cycle, we tested the impact of IDH2 knockdown on the cellular level of α-KG in TNBC cells, using specific shRNA against IDH2 and scrambled RNA vector as the control. Surprisingly, we observed a significant increase in cellular α-KG after IDH2 knockdown in MDA-MB-231 cells (Fig. 2a). This was unexpected since the inhibition of IDH2 would normally lead to a decrease in α-KG based on the metabolic flow of the TCA cycle in the oxidative direction. To further confirm this unexpected result, we used another TNBC cell line (HCC38) for IDH2 knockdown, and observed a similar increase of α-KG (Fig. 2b). Importantly, analysis of α-KG levels in tumor tissues isolated from mice inoculated with MDA-MB-231 cells with or without IDH2 knockdown revealed an approximately threefold increase of α-KG in the tumor tissues with IDH2 silencing (Fig. 2c), indicating that the change in α-KG was consistent in vitro and in vivo.

The above observations prompted us to investigate the potential mechanisms by which IDH2 abrogation caused α-KG elevation. We used [U^13^C]-labeled glutamine as a tracing agent to analyze the metabolic flow in the IDH2-inhibited cells in comparison with the control cells harboring an empty vector, as illustrated in Fig. 2d. GC-MS analysis revealed that there was a significant increase in glutamine uptake (Fig. 2e) and elevated intracellular glutamate (Fig. 2f) in the IDH2-knockdown cells. Gene expression analysis showed that phosphoserine aminotransferase 1 (PSAT1), an enzyme that converts phosphohydroxypyruvate (PHP) to phosphoserine (pSer) coupled with generation of α-KG from glutamate, was significantly elevated (Fig. 2g). The ratio of Serine/PEP substantially increased in the IDH2-knockdown cells (Fig. 2h), consistent with high PSAT1 enzyme activity. These data together show that the metabolic disturbance induced by IDH2 abrogation led to elevated α-KG due to an increase in glutamine uptake and its subsequent conversion to α-KG by PSAT1, a molecule known to respond to metabolic stress via NRF2 and ATF4^24^. Consistently, this metabolic flux analysis also revealed an increase in α-KG abundance using the GC-MS method (Fig. 2i) and in MPE (mole percent enrichment) of α-KG conversion from glutamine (Fig. 2j and Supplementary Fig. S3a). Importantly, we found that more than 50% of α-KG was derived from glutamine in TNBC cells, as indicated by the MPE value of 0.51 in the control MDA-MB-231 cells (Fig. 2j), suggesting an active metabolic flow from glutamate to α-KG feeding into the TCA cycle in TNBC cells. Consistently, there was a significant decrease in the molar ratios of isocitrate/α-KG and citrate/α-KG in the IDH2-knockdown cells (Fig. 2k).

Quantitation of the TCA cycle metabolites in cells labeled with [U^13^C]-glutamine showed an increase in glutamine→α-KG (M + 5) flux (Supplementary Fig. S3a) and an accumulation of succinate (M + 4) with IDH2 KD (Supplementary Fig. S3b), suggesting that a knockdown of IDH2 did not block the forward flux from α-KG to succinate. Interestingly, the labeled citrate/isocitrate (M + 4) decreased with IDH2 KD (Supplementary Fig. S3c, d), indicating that the forward flux from (M + 4) succinate to citrate/isocitrate was suppressed. There were no significant changes in other metabolites' TCA cycle and glycolysis (Supplementary Fig. S3e–h). We noted that there was little M + 5 citrate/isocitrate detectable in MDA-MB-231 cells labeled with [U^13^C]-glutamine (Supplementary Fig. S3c, d), which appeared inconsistent with high reductive TCA metabolism and thus needed further testing as described below.

Since the metabolic flux from [U^13^C]-glutamine to M + 5 (iso)citrate could be highly dynamic, with M + 5 (iso)citrate being constantly transported to cytosol, where it is converted to acetyl-CoA (Ac-CoA) for de novo fatty acid synthesis. This would make it necessary to determine the flux of M + 5 (iso)citrate to ^13^C-labeled fatty acids in order to fully assess the reductive TCA activity. As shown in Supplementary Fig. S4a, the lipogenic Ac-CoA from M + 5 (iso)citrate via reductive TCA should contain M + 2 carbons, whereas the Ac-CoA from M + 4 (iso)citrate through oxidative TCA would only contain unlabeled carbons. This difference in ^13^C-labeled fatty acids enabled us to differentiate the reductive metabolic reflux from the oxidative TCA according to the previously published method^18^. Thus, we tested the flux of [U^13^C]glutamine to ^13^C-labeled palmitate in MDA-MB-231 cells and the impact of the IDH2 inhibitor AGI-6780, which is known to bind IDH2 at the dimer interface and allosterically interferes with the dimerization of the enzyme^25^, on this metabolic process. The relative abundances of the labeled metabolites are shown in Supplementary Fig. S4b–i. We observed an increase of labeled α-KG (M + 5) when IDH2 was inhibited (Supplementary Fig. S4b). The levels of the labeled citrate and isocitrate (M + 5) were again very low (Supplementary Fig. S4c, d), similar to that observed in Supplementary Fig S3c, d. However, analysis of [U^13^C]-glutamine flux to palmitate revealed a 7.2% (relative abundance) of ^13^C-labeled carbon in the newly synthesized palmitate, indicating an active reductive TCA metabolism in TNBC cells (Supplementary Fig. S4e). Importantly, inhibition of IDH2 by AGI-6780 resulted in a 45% decrease in [U^13^C]-glutamine flux to palmitate (from 7.2 to 3.9%, Supplementary Fig. S4e), indicating a significant role of IDH2 in mediating the reductive TCA metabolism to utilize glutamine for lipid synthesis. Interestingly, we also observed that some of the (M + 4)-labeled metabolites downstream of succinate, such as fumarate and malate, were decreased when IDH2 was inhibited by AGI-6780 (Supplementary Fig. S4f–h), suggesting that the forward flow of the TCA cycle was so suppressed. Very little labeled pyruvate or PEP were detected in TNBC cells (Supplementary Fig. S4i, j). When cells were treated with AGI-6780, the relative levels of isocitrate and citrate calculated as MPE/α-KG were decreased by 69 and 31%, respectively (Supplementary Fig. S4k).

We repeated the glutamine flux experiment, using a new batch of [U^13^C]-glutamine with high labeling efficiency. The results of the second experiments (Supplementary Fig. S5 and Supplementary Data Files 1–3) were very similar to that of the first flux experiment, consistently showing that AGI-6780 caused an increase in (M + 5) α-KG (Supplementary Fig. S5a), a decrease in (M + 4) isocitrate and a relative increase in (M + 5) isocitrate (Supplementary Fig. S5b). Similar changes were also observed with citrate (Supplementary Fig. S5c). The impacts of IDH2 inhibition by AGI-6780 on glutamine flux of to various other metabolites are shown in (Supplementary Figs. S5d–k). Of note, the inhibitory effect of AGI-6780 on newly synthesized palmitate was substantial, evidenced by a significant decrease of U^13^C-labeled palmitate from 19% in the control cells to 12.8% in the AGI-treated cells (Supplementary Fig. S5e), representing a 33% inhibition. Also, the 19% [^13^C]-glutamine flux to palmitate in the control cells suggests a very active reductive TCA metabolism in TNBC cells. Interestingly, AGI-6780 also potently inhibited U^13^C-glutamine flux to succinate by 68%, as estimated by the decrease of U^13^C-labeled succinate (Supplementary Fig. S5f and Supplementary Data File 1), suggesting a strong inhibition of oxidative TCA metabolism by AGI-6780. To further confirm this inhibition, we used [U^13^C]-labeled glucose as another tracing agent, which would be converted to [^13^C]-pyruvate to analyze the oxidative TCA metabolic flow in MDA-MB-231 cells treated with AGI-6780 or with solvent control (Supplementary Fig. S6a). GC-MS analysis of M + 2 & M + 4 labeled citrate and its oxidative metabolites (M + 2 and M + 4 labeled succinate, fumarate, and malate) showed that AGI-6780 inhibited oxidative TCA metabolism by approximately 60–80% (Supplementary Fig. S6b–f and Supplementary Data File 4). This degree of inhibition was similar to the 68% inhibition observed in the [U^13^C]-glutamine tracing experiment.

A rescue experiment using an IDH2 expression vector resistant to shRNA-#2 in TNBC cells with pre-knockdown of IDH2 showed that the re-expression of IDH2 could reduce the intracellular α-KG level to the control (NC) level (Supplementary Fig. S7a, b), indicating that the changes in α-KG levels was specifically due to the change in IDH2, not due to the off-target effect in the knockdown experiment. We also compared the α-KG levels in MDA-MB-231 cells with that of another TNBC cell line (HCC38) with higher IDH2 expression, and found that the basal α-KG level in HCC38 cells was slightly higher than that of MDA-MB-231 cells (Supplementary Fig. S7c). The level of α-KG was substantially lower in the non-cancer cells (MCF10A), which also exhibited lower expression of IDH2 (Supplementary Fig. S7c).

### Accumulation of α-KG induced by IDH2 abrogation is detrimental to TNBC cells by double-hit on energy metabolism

**Fig. 3:**
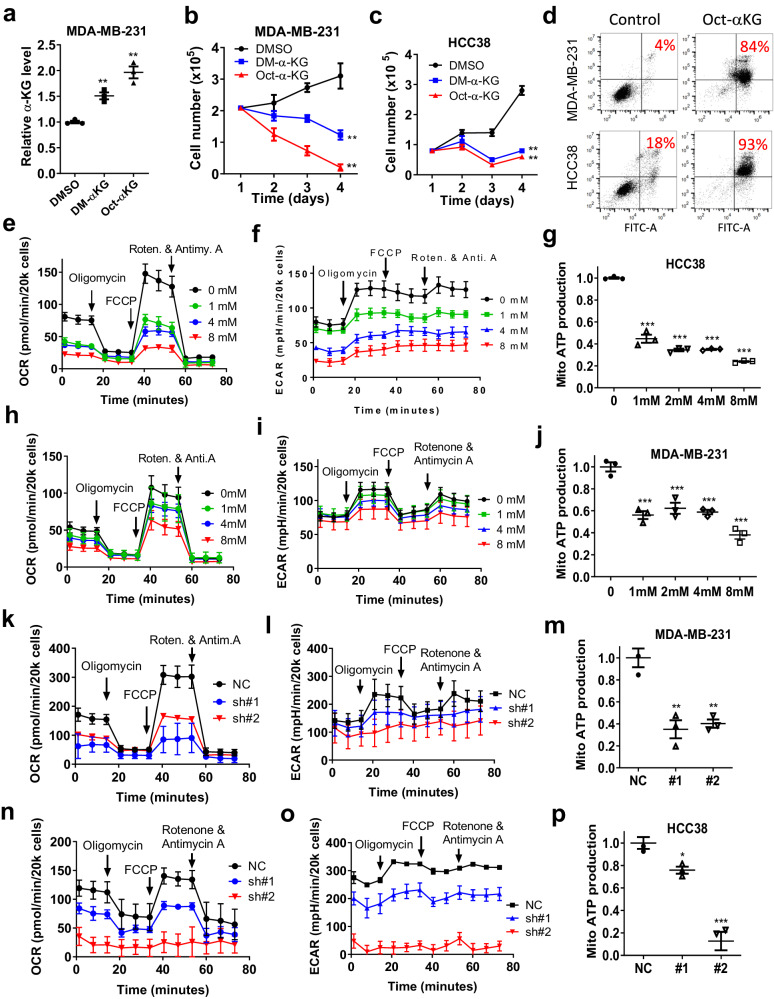
Accumulation of α-KG induced by IDH2 abrogation was detrimental to TNBC cells due to double-hit on energy metabolism. **a** Incubation of MDA-MB-231 cells with cell-permeable DM-α-KG or Oct-α-KG (1 mM) led to an increase in cellular α-KG level (*n* = 3, technical replicates, representative of two independent experiments, original data included in the Source Data). **b**, **c** Effect of DM-α-KG (1 mM) or Oct-α-KG (1 mM) on cell viability in MDA-MB-231 or HCC38 cells (*n* = 3 each, technical replicates, representative of two independent experiments, original data included in the Source Data). The numbers of viable cells were determined using a Trypan blue exclusion assay. **d** Flow cytometer analysis of MDA-MB-231 or HCC38 cells treated with Oct-α-KG (2 mM, 48 h) using Annexin V-FITC/PI apoptosis assay. The number within each panel shows % viable cells. **e**–**g** Oxygen consumption rate (OCR, **e**), extracellular acidification rate (ECAR, **f**), and the calculated mitochondrial ATP production rate (**g**) in HCC38 incubated with cell-permeable DM-α-KG for 12 h. OCR and ECAR were measured using the Seahorse metabolic analyzer. **h**–**j** Effect of DM-α-KG on OCR (**h**), ECAR (**i**), the calculated mitochondrial ATP production rate (**j**) in MDA-MB-231 cells (12 h incubation). **k**–**m** Effect of IDH2 knockdown by shRNA (#1 and #2) on OCR (**k**), ECAR (**l**), and the calculated mitochondrial ATP production (**m**) in MDA-MB-231 cells. **n**–**p** Effect of IDH2 knockdown by shRNA (#1 and #2) on OCR (**n**), ECAR (**o**), and the calculated mitochondrial ATP production (**p**) in HCC38 cells. Error bars in all panels represent mean values ± S.D. (*n* = 3 for each group in **e**–**p**, technical replicates). A two-sided Student’s *t*-test was used for statistical analysis. *p ≤ 0.05; **p ≤ 0.01; ***p ≤ 0.001.

Since the knockdown of IDH2 caused a significant increase of α-KG in TNBC cells, we thus investigated the direct impact of α-KG on cancer cell growth and viability using multiple assays. First, TNBC cells were incubated with cell-permeable dimethyl-α-KG or octyl-α**-**KG, and cell growth was measured by direct cell counting. We observed that the addition of exogenous dimethyl-α-KG or octyl-α**-**KG to cell culture caused a significant increase of intracellular α-KG (Fig. 3a), and dramatically reduced the growth of MDA-MB-231 cells (Fig. 3b). The inhibition of cell growth by cell-permeable α-KG was also observed in another TNBC cell line HCC38 (Fig. 3c). Flow cytometry analysis showed that dimethyl-α-KG and octyl-α**-**KG induced massive apoptosis in both MDA-MD-231 and HCC38 cells (Fig. 3d and Supplementary S8a, b). Although the concentrations of cell-permeable dimethyl-α-KG or octyl-α**-**KG were at mM range, the intracellular α-KG increased to 1.5–2.0 folds of the control cells (Fig. 3a), which was similar to the α-KG increase in the IDH2-knockdown cells (Fig. 2a, b), suggesting that the impact of dimethyl-α-KG or octyl-α**-**KG on intracellular α-KG was in a physiological range.

We then focused on the potential mechanisms by which α-KG might affect TNBC cell viability and tumor growth. Based on the ability of α-KG to inhibit ATP synthase^26^ and to suppress glycolysis observed in our recent study^19^, we speculated that a high level of α-KG in TNBC cells might impact both mitochondrial respiration and glycolysis, leading to a severe ATP depletion and loss of cell viability. Indeed, treatment of TNBC cells (HCC38) with dimethyl-α-KG caused a concentration-dependent suppression of oxygen consumption rate (OCR, Fig. 3e) and a reduction of extracellular acidification rate (ECAR, Fig. 3f), accompanied by a significant decrease in ATP generation by mitochondria (Fig. 3g). Measurement of cellular ATP contents consistently showed a significant decrease in HCC38 cells after treatment with dimethyl-α-KG (Supplementary Fig. S8c). Similar results were also observed in MDA-MB-231 cells (Fig. 3h–j and Supplementary Fig. S8d). At this early time point, no significant cell death was detected (Supplementary Fig. S8e). Since knocking down of IDH2 in TNBC cells resulted in an increase of cellular α-KG, we hypothesized that such knockdown would also cause a decrease in OCR and ECAR. Indeed, silencing IDH2 expression in MDA-231 cells by shRNA led to a substantial decrease in OCR and ECAR (Fig. 3k, l), with a ~65% decrease in mitochondrial ATP generation (Fig. 3m). Similar results were also observed in HCC38 cells transfected with IDH2-shRNA (Fig. 3n–p). These data together suggest that the abnormal increase in α-KG induced by IDH2 abrogation in TNBC cells caused a double inhibition of glycolysis and oxidative phosphorylation, leading to ATP depletion and cell death.

### IDH2 enhances HIF-1α stabilization and promotes TNBC metastasis

**Fig. 4:**
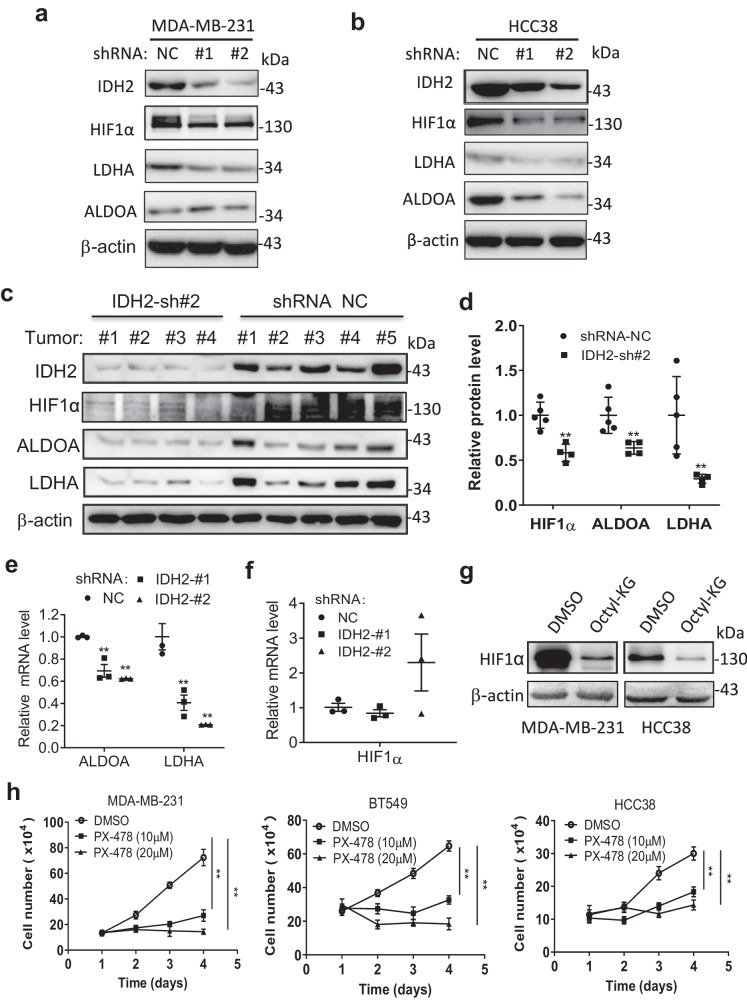
IDH2 promotes HIF-1α protein stability through modulation of α-KG. **a**, **b** Western blot analysis of IDH2, HIF1α and its downstream molecules in MDA-MB-231 and HCC38 cells transfected with IDH2 shRNA (#1 and #2) or control RNA (NC). The samples were derived from the same experiment, but different gels (one gel for IDH2 and ALDOA, another for LDHA, another for HIF1α, and another for β-actin) were processed in parallel. Images are representative of two independent experiments (original data included in the Source Data). **c**, **d** Western blot analysis of IDH2, HIF1α, ALDOA, and LDHA in tumor tissues from mice inoculated with MDA-MB-231 cells transfected with IDH2 shRNA (sh#2, *n* = 4, independent biological replicates) or control shRNA (NC, *n* = 5, independent biological replicates). The relative protein levels were quantified and normalized by β-actin. The samples were derived from the same experiment, but different gels (one gel for IDH2, ALDOA, and LDHA, another gel for HIF1α and β-actin) were processed in parallel (*n* = 5 for the NC group, *n* = 4 for the shRNA group, independent biological replicates). **e**, **f** Relative mRNA levels of ALDOA and LDHA (**e**) and HIF1α (**f**) in MDA-MB-231 cells transfected with IDH2 shRNA (#1 & #2) or control RNA (NC), *n* = 3 (independent experiments). **g** Western blot analysis of HIF1α expression in MDA-MB-231 or HCC38 cells cultured with octyl-α-KG (1 mM, 48 h). β-actin was run in the same gel as a loading control. images are representative of two independent experiments. **h** Effect of HIF1α inhibitor (PX-478) on cell viability in three TNBC cell lines, measured by Trypan blue exclusion assay. Error bars in all panels represent the mean values ± S.D., *n* = 3 (technical replicates, representative of two independent experiments, original data included in the Source Data). A two-sided Student’s *t*-test was used for statistical analysis (*p ≤ 0.05; **p ≤ 0.01; ***p ≤ 0.001).

Since HIF1α is known to promote the expression of several key glycolytic enzymes and α-KG could affect HIF1α stability via prolyl hydroxylase domain-containing protein^27–29^, we hypothesized that IDH2 might impact HIF1α and glycolysis by altering α-KG level in TNBC cells. To explore this possibility, we first used the public datasets to examine the potential relationship between IDH2 expression and HIF1α signature in TNBC cells. We found that IDH2 expression was significantly correlated with the overall HIF1α signature expression (Supplementary Fig. S9a) and other HIF1α target molecules (Supplementary Fig. S9b). We then tested the cause-effect relationship between IDH2 expression and HIF1α. As shown in Fig. 4a, b, knocking down of IDH2 using shRNA in TNBC cells resulted in a substantial decrease in HIF1α protein and its downstream molecules ALDOA and LDHA. Furthermore, analysis of protein extracts of the tumor tissues from mice inoculated with MDA-MB-231 cells with IDH2-knockdown showed that the protein level of HIF1α and its target genes LDHA and ALDOA were substantially decreased (Fig. 4c, d), indicating that IDH2 had a significant impact on HIF1a and its downstream molecules in vitro and in vivo.

Analysis of mRNA expression revealed that a knockdown of IDH2 led to a significant decrease in ALDOA and LDHA mRNA (Fig. 4e) but not in HIF1α mRNA (Fig. 4f), suggesting that IDH2 mainly regulated the stability of HIF1a protein, which in turn affect the expression of its downstream target genes. To further demonstrate the effect of IDH2 on HIF1α stability, we tested the effect of IDH2 inhibition by AGI-6780 on HIF1α stability in NTBC cells, using a time-course experiment after cycloheximide (CHX) blockage. The results consistently showed that IDH2 inhibition accelerated HIF1α protein degradation (Supplementary Fig. S9c, d), and led to a decrease in expression of HIF1α target genes LDHA and ALDOA (Supplementary Fig. S9e). To further test the contribution of HIF1α to the effects of IDH2 inhibition, we performed a HIF1α “rescue” experiment by using two different inhibitors of prolyl hydroxylase (TP0463518 and IOX4) to stabilize HIF1α in TNBC cells treated with AGI-6780. We found that the stabilization of the HIF1α protein by the prolyl hydroxylase inhibitors could significantly reverse the suppressive effect of IDH2 inhibition on cell proliferation (Supplementary Fig. S9f).

We then tested the impact of IDH2 overexpression on the cellular level of α-ketoglutarate, HIF1α, and cell proliferation in TNBC cells (MDA-MB-231 and BT474), using two IDH2 expression vectors. As shown in Supplementary Fig S9g–i, IDH2 overexpression caused a moderate but statistically significant decrease of intracellular α-ketoglutarate content in both cell lines, which also exhibited a modest increase in HIF1α level. The overexpression of IDH2 also resulted in a moderate but statistically significant increase in cell proliferation (Supplementary Fig. S9j, k).

Since knocking down of IDH2 caused a significant increase in α-KG (Fig. 2a–c), we then tested the direct effect of α-KG on HIF1α. As shown in Fig. 4g, treatment of MDA-MB-231 or HCC38 cells with cell-permeable octyl-α-KG caused a major decrease of HIF1α protein in both cell lines. HIF1α is known to regulate multiple glycolytic enzymes, the decrease of HIF1α protein induced by α-KG could explain why IDH2 knockdown led to an inhibition of glycolysis. To further validate the important role of HIF1α in TNBC cells, we used the HIF1α inhibitor PX-478 to test its impact on TNBC cells. As shown in Fig. 4h, PX-478 potently inhibited the proliferation of three TNBC cell lines (MDA-MB-231, HCC38, and BT549). These data together suggest that suppression of IDH2 impacts TNBC cells in part via the α-KG/HIF1α axis.

**Fig. 5:**
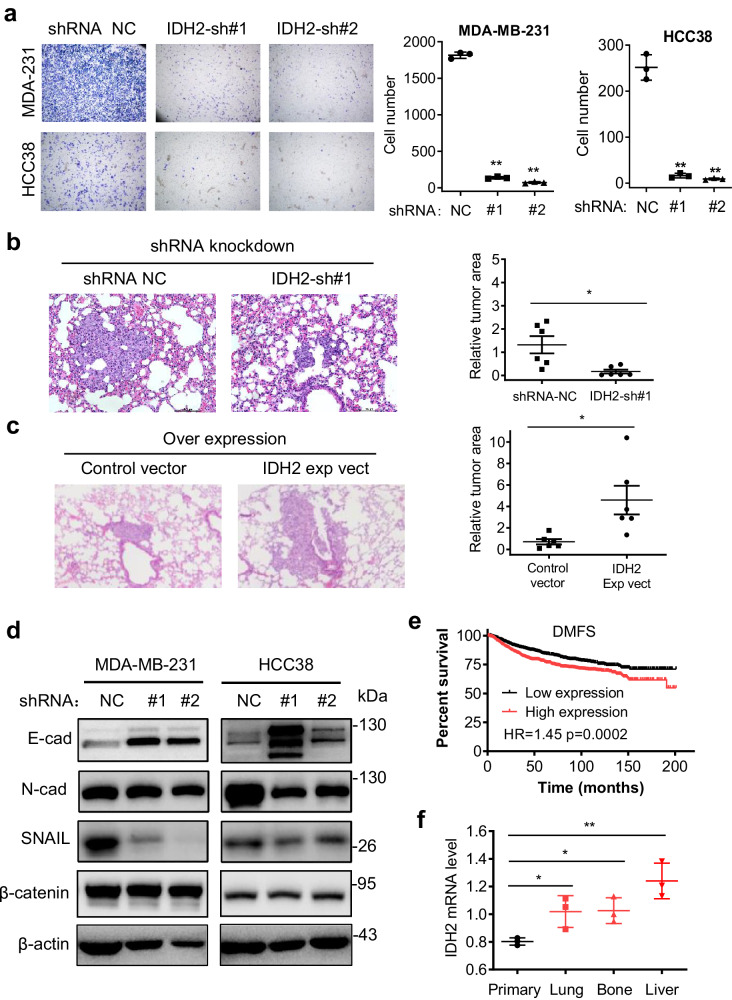
Impact of IDH2 on TNBC cell migration in culture and tumor metastasis in mice. **a** Comparison of cell migration in TNBC cells transfected with IDH2 shRNAs (#1, #2) or with non-targeting control shRNA (NC). Cell migration was measured by a transwell assay (24 h). The graphs show the representative data of three separate measurements. **b**, **c** Hematoxylin-Eosin (HE) staining of the lung tissues from nude mice injected (via tail veins) with MDA-MB-231 cells transfected with IDH2 shRNA (#1) or with non-targeting control RNA (NC) (**b**, *n* = 6, independent biological replicates), or with IDH2-overexpression vector (**c**, *n* = 6, independent biological replicates). Tumor areas in the stained lung tissues were quantified using Image J software (right panels). **d** Western blot analysis of the key EMT-related proteins in TNBC cell lines transfected with IDH2 shRNA (#1) or with non-targeting control RNA (NC); images are representative of two independent experiments (original data included in the Source Data). The samples were derived from the same experiment, but different gels (one gel for N-cad, Snail, and β-actin, another for β-catenin, and another for E-cad) were processed in parallel. **e** Kaplan–Meier survival curves of breast cancer patients stratified by IDH2 expression. The total number of samples analyzed was 1722 for distant metastasis-free survival; the median value was used as the cut-off point (Kmplot). **f** Comparison of IDH2 mRNA levels in primary tumors and metastatic tumors in lung, bone, or liver isolated from mice inoculated with 4T1 breast cancer cell line (analysis of dataset from GEO, GSE62598). Error bars in all panels of represent mean values ± S.D. (*n* = 3, independent biological replicates); a two-sided Student’s *t*-test was used for statistical analysis. *p ≤ 0.05; **p ≤ 0.01.

Because HIF1α is also known to promote metastasis in breast cancer cells^30–32^, For metastasis genes, snail is one of the key genes to promote EMT and metastasis progress, which is upregulated by HIF1α^33^. We thus tested if IDH2 might affect TNBC metastasis using in vitro and in vivo models. Both transwell cell invasion and wound-healing assays showed that a knockdown of IDH2 significantly suppressed TNBC cell invasion (Fig. 5a) and decreased cell migration (Supplementary Fig. S10a). Importantly, in vivo experiments using TNBC cells with stable knock-down or overexpression of IDH2 in a lung colonization model showed that IDH2 knock-down led to significantly less and smaller tumor metastatic nodules in the lungs (Fig. 5b and Supplementary Fig. S10b), while overexpression of IDH2 resulted in a major increase in lung metastatic tumor nodules (Fig. 5c and Supplementary Fig. S10c). Since the epithelial-to-mesenchymal transition (EMT) process is closely correlated with cancer cell migration and metastasis^34^, we analyzed the expression of EMT-related genes in IDH2 knock-down TNBC cell lines, and found that E-cadherin expression was significantly increased, whereas SNAIL expression was substantially decreased in the IDH2 knockdown cell lines (Fig. 5d). The expression of N-cadherin also decreased in MDA-MB-231 after knockdown of IDH2 (Fig. 5d). IHC analysis of E-cadherin expression in metastasis site show higher expression of IDH2 in knockdown cell (Supplementary S10d, e). None of the mice died at the end of the experiments. The animals were terminated for pathological analysis of lung metastasis 113 days after tail-vain tumor cell injection. We also performed a rescue experiment by constructing an IDH2 expression vector specifically resistant to IDH2 shRNA #2 in two TNBC cell lines (HCC38 and MDA-MD-231) that had been pre-knocked down with IDH2 shRNA #2. The results showed that re-expression of IDH2 largely restored the expression of E-cadherin protein expression (Supplementary Fig. S10f). These results together suggest that wild-type IDH2 might play a significant role in promoting EMT and metastasis in TNBC. Consistently, analysis of IDH2 expression in the GEO database revealed that high IDH2 mRNA level was correlated with worse distant metastasis-free survival (Fig. 5e) and that IDH2 mRNA expression was upregulated in metastasized TNBC cells (Fig. 5f).

### Pharmacological inhibition of IDH2 is effective against TNBC in vitro and in vivo

**Fig. 6:**
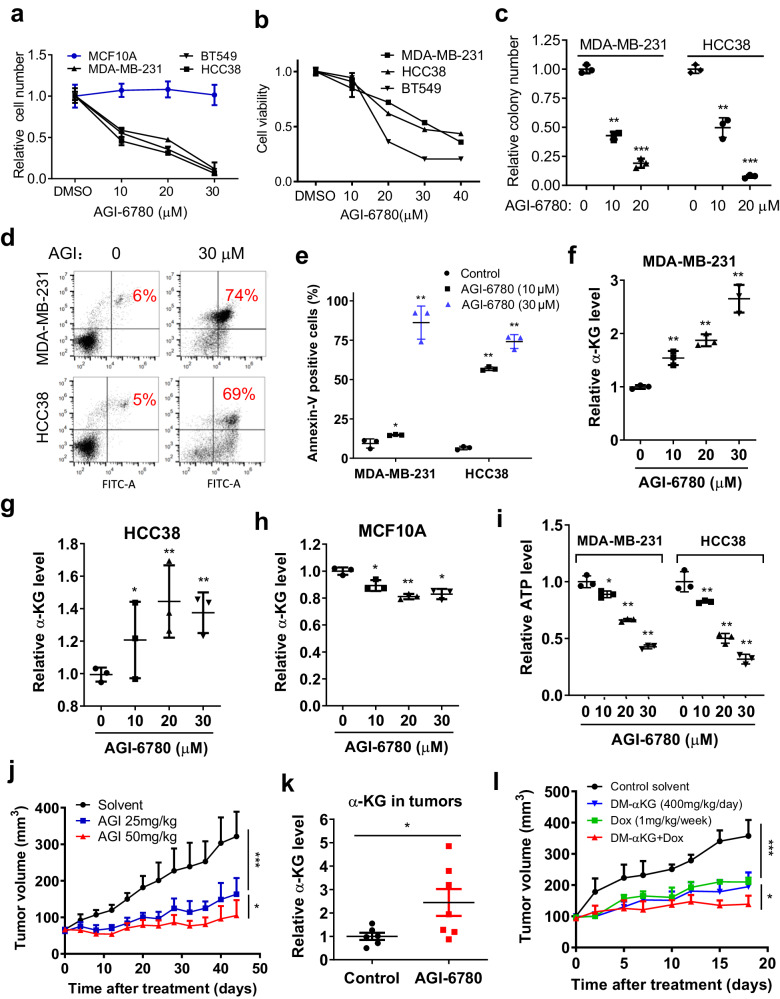
Therapeutic effect of IDH2 inhibitor AGI-6780 against TNBC in vitro and in vivo. **a** Effect of AGI-6780 on the proliferation of TNBC cells and MCF10A breast epithelial cells. Cell proliferation was measured by direct cell counting at 48 h, *n* = 3 for each group (technical replicates, representative of two independent experiments, original data included in the Source Data). **b** Effect of AGI-6780 on TNBC cell viability measured by MTS assay at 48 h, *n* = 3 for each group (technical replicates, representative of two independent experiments (original data included in the Source Data). **c** Impact of AGI-6780 on the ability of TNBC cells to form colonies (14 days), *n* = 3 for each group (technical replicates, representative of two independent experiments, original data included in the Source Data). **d**, **e** Flow cytometer analysis of MDA-MB-231 or HCC38 cells treated with or without AGI-6780 for 48 h. Apoptotic cells were detected using Annexin V-FITC/PI double staining, *n* = 3 for each group (technical replicates, representative of two independent experiments, original data included in the Source Data). **f**–**h** Effect of AGI-6780 on cellular α-KG levels in TNBC cell lines (**f**, **g**) or MCF10A breast epithelial cells (**h**). **i** Relative cellular ATP levels in TNBC cell lines before and after treatment with the indicated concentrations of AGI-6780 for 12 h, *n* = 3 (technical replicates) for each group. **j** In vivo therapeutic effect of AGI-6780 on tumor growth in mice inoculated with MDA-MB-231 cells. The indicated doses of AGI-6780 were injected intraperitoneally. DMSO was diluted in PBS as solvent control for i.p. injection into the control mice, *n* = 6 for the DMSO group, and *n* = 8 mice for the AGI-6780 treated group, two-way ANOVA test was employed for statistical analysis, *p ≤ 0.05; **p ≤ 0.01, ***p ≤ 0.001. **k** Relative α-KG levels in MDA-MB-231 xenograft tumor tissues from mice treated with AGI-6780 (50 mg/kg) or with solvent control (control: *n* = 6, AGI-treated: *n* = 7, independent biological replicates). **l** Mice bearing MDA-MB-231 xenografts were treated by intra-peritoneal injection with solvent (diluted DMSO), dimethyl-α-KG, doxorubicin, or the combination of dimethyl-α-KG and doxorubicin as indicated. Tumor sizes were measured every 2−3 days, *n* = 6 mice for each group, two-way ANOVA test was employed for statistical analysis, *p ≤ 0.05; **p ≤ 0.01, ***p ≤ 0.001. Error bars in all panels represent mean values ± S.D. Two-sided Student’s *t*-test was used for statistical analysis, *p ≤ 0.05; **p ≤ 0.01.

Considering the critical role of wt-IDH2 in TNBC cell survival and tumor growth and metastasis, we reasoned that pharmacological inhibition of wt-IDH2 would be effective against TNBC. To test this possibility, we used AGI-6780 (*N*-Cyclopropyl-4-(3-thienyl)-3- [[[[3-(trifluoromethyl)phenyl]amino]carbonyl]amino]-benzenesulfonamide), an inhibitor of mutant and wild-type IDH2^25^, to evaluate its impact on multiple TNBC cell lines harboring wt-IDH2. As shown in Fig. 6a, incubation of MDA-MB0231, HCC38, or BT549 cells with AGI-6780 significantly suppressed cell growth, as evidenced by a concentration-dependent reduction of cell number. Interestingly, parallel experiment showed that the non-tumorigenic mammary epithelial cells (MCF10A) were insensitive to AGI-6780 (Fig. 6a). Cell survival and colony formation assays also showed that AGI-6780 significantly reduced TNBC cell viability (Fig. 6b) and their ability to form colonies (Fig. 6c). Flow cytometry analysis demonstrated that inhibition of IDH2 by AGI-6780 caused a massive apoptosis in TNBC cells, as evidenced by the appearance of annexin V-positive cells (Fig. 6d, e).

Biochemically, we found that inhibition of IDH2 by AGI-6780 caused a significant increase of α-KG in MDA-MB-231 cells (Fig. 6f) and HCC38 cells (Fig. 6g), consistent with the observed change of α-KG in the IDH2-knockdown TNBC cells (Fig. 2a–c). RT-PCR analysis of mRNA expression revealed that among the genes involved in α-KG metabolism, PSAT1 was particularly upregulated in TNBC cells after IDH2 was inhibited by AGI-6780 (Supplementary Fig. S11). This was similar to the elevated PSAT1 expression observed in the IDH2- knockdown cells (Fig. 2g), and again suggests an active conversion of glutamate to α-KG for replenishing the reductive TCA cycle in TNBC cells. Interestingly, treatment of non-tumorigenic MCF10A mammary cells with AGI-6780 did not cause elevation of α-KG, and a moderate decrease was detected instead (Fig. 6h), suggesting a major difference in the metabolic flows (oxidative or reductive) of the TCA cycle between TNBC cells and the non-tumorigenic mammary epithelial cells. This might explain why MCF10A cells were not sensitive to AGI-6780.

The ability of AGI-6780 to induce accumulation of α-KG in TNBC cells prompted us to test its impact on cellular ATP, based on the observations that elevated α-KG could inhibit both OCR and ECAR (Fig. 3). As expected, treatment of MDA-MB-231 or HCC38 cells with AGI-6780 resulted in a concentration-dependent depletion of ATP in both cell lines (Fig. 6i).

To assess the in vivo therapeutic activity of AGI-6780 against TNBC, we tested the effects of AGI-6780 in a xenograft tumor model. MDA-MB-231 cells were injected orthotopically into the mammary glands of nude mice. After the tumors reached 5–6 mm in diameter, the mice were randomly divided into three groups for treatment with (1) Control solvent (diluted DMSO); (2) AGI-6780 25 mg/kg/day; (3) AGI-6780 50 mg/kg/day. As shown in Fig. 6j, AGI-6780 treatment significantly suppressed tumor growth in a dose-dependent manner. Importantly, analysis of α-KG in the tumor tissues isolated from the control or the drug-treated mice showed that AGI-6780 could cause a significant increase of α-KG in vivo (Fig. 6k), indicating that the biochemical mechanism identified in vitro was also seen in vivo. We then tested if the elevated α-KG could mediate the therapeutic effect of IDH2 inhibition in animals, using the cell-permeable compound DM-α-KG for the treatment of mice bearing MDA-MB-231 tumor xenografts. As shown in Fig. 6i, DM-α-KG at a dose of 400 mg/kg exhibited significant therapeutic activity in suppressing tumor growth. This therapeutic effect could be further potentiated by combination with chemotherapeutic drug doxorubicin (Fig. 6i). The mechanism for this potentiation of anticancer effect was likely due to DM-α-KG-induced depletion of ATP, which could then possibly reduce the ability of ATP-dependent pump to export doxorubicin from the tumor cells, leading to more accumulation of doxorubicin in TNBC cells (Supplementary Fig. S12). These results together showed that targeting IDH2 or direct modulation of α-KG might be an effective treatment strategy for TNBC.

## Discussion

Breast cancer is a leading cause of cancer-related deaths in women^35^, and can be classified, according to their molecular characteristics and gene expression profiles, into different subgroups that are associated with different outcomes and require different treatment regimens^36^. Among these subtypes, triple-negative breast cancer is an aggressive subgroup with the cancer cells lacking the expression of estrogen receptor (ER), progesterone receptor (PR), and human epidermal growth factor receptor 2 (HER2)^37^. Due to the absence of ER/PR receptors and the ability to proliferate autonomously without the need for extracellular hormone stimulation, TNBC cells exhibit aggressive malignant behaviors, and in general, are not sensitive to hormonal therapy or to drugs that target HER2 such as trastuzumab (Herceptin) and pertuzumab (Perjeta). As such, patients with TNBC are usually prone to develop higher-grade and invasive tumors with poor clinical outcome^38–40^. Currently, there are limited treatment options for TNBC. Conventional chemotherapeutic agents such as doxorubicin, cyclophosphamide, and paclitaxel are used to treat this subtype of breast cancer^41^. The results of such chemotherapy are often unsatisfactory, and many TNBC patients eventually exhibit relapse and metastasis^42,43^. Thus, it is important to explore new therapeutic strategies for the treatment of TNBC based on their biological characteristics, such as alterations in certain molecular signaling and changes in metabolic pathways.

Our previous study showed that TNBC exhibited profound metabolic alterations manifested by decreased mitochondrial respiration and increased glycolysis^44^, suggesting that it might be possible to target the metabolic alterations in TNBC cells as a new therapeutic strategy. However, it remains unclear how TNBC cells maintain their high glycolytic activity and what would be the effective strategy to target the key mechanism that supports the metabolic alterations in TNBC cells. In the present study, we showed that a high basal level of HIF-1α protein seemed important for TNBC cell viability due to the essential role of HIF-1α in maintaining active glycolysis, and that a timely conversion of α-KG to isocitrate through the reductive TCA cycle seems critical for the stability of HIF-1α in TNBC cells. As such, IDH2 is particularly important for TNBC cells due to its critical role in catalyzing the reductive conversion of α-KG to isocitrate. Thus, inhibition of IDH2 would lead to an abnormal accumulation of α-KG that, in turn, promoted the degradation of HIF-1α, leading to suppression of glycolysis. A similar phenomenon could also be found in lung cancer cell^19^. However, opposite results have been seen in other epithelial cancers, as evidenced by the findings that IDH2 knockdown could increase HIF protein through increased ROS generation in prostate cancer cells^45^, These observations suggest that the effect of IDH2 on HIF could be cell-type dependent, likely due to different intrinsic metabolic characteristics in different cell types. The high expression of IDH2 and its effect on HIF seem particularly important for TNBC cells, since the high IDH2 enzyme activity is required for timely conversion of α-KG to isocitrate/citrate. This would enable the TNBC cells to keep α-KG at a relatively low level to prevent HIF protein degradation, and at the same time, generate more citrate for lipid synthesis.

The conversion of α-KG to isocitrate catalyzed by IDH2 in the reductive TCA cycle seems particularly important for TNBC cells due to their active utilization of glutamine channeled to reductive TCA cycle that generates citrate for lipid synthesis, a metabolic property of TNBC revealed in our metabolic flow analysis and gene expression assay (Fig. 2). The evidence for active glutamine utilization in TNBC cells include: (a) a high uptake of glutamine; (b) the active metabolic flow from glutamine to glutamate and then to α-KG; (c) a high expression of PSAT1 that converts glutamate to α-KG; and (d) a significant elevation of α-KG when IDH2 was inhibited. These data also suggest that the metabolic flow from α-KG to isocitrate/citrate in the reductive TCA cycle is intrinsically active in TNBC cells, probably due to a need to generate citrate as an important metabolic precursor for lipid synthesis to support the cancer cell proliferation. As such, a high expression of IDH2 is particularly important for TNBC cells to ensure a timely conversion of α-KG to isocitrate, and suppression of IDH2 by siRNA or chemical inhibition would lead to high accumulation of α-KG in TNBC cells.

Of note, the [U^13^C]-glutamine flux experiments consistently showed a significant increase of (m + 5) α-KG in cells treated with the IDH2 inhibitor AGI-6780 (Figs. S4b, S5a), suggesting that the reductive TCA metabolism was inhibited. However, AGI-6780 also caused an increase in (m + 5) isocitrate and (m + 5) citrate with a simultaneous decrease in isocitrate and citrate, (m + 4) isocitrate and (m + 4) citrate (Figs. S4c, d,  S5b, c), which appears contradictory to the above conclusion. These seemingly paradoxical results were consistently observed in separate experiments, and require mechanistic exploration. One possibility could be that AGI-6780, as a chemical inhibitor of IDH2, could suppress both reductive and oxidative TCA metabolism at the step of isocitrate/α-KG interconversion, and thus could exert a strong inhibition on the oxidative reaction (isocitrate → α-KG) and a less potent inhibition on the reductive metabolism (α-KG → isocitrate). This uneven inhibition on different metabolic directions would lead to a low proportion of (m + 4)-labeled isocitrate from oxidative TCA metabolism and a relatively higher fraction of (m + 5)-labeled isocitrate as the reductive products due to a stronger inhibition on oxidative TCA flow and less inhibition on reductive TCA. Indeed, we observed that AGI-6780 inhibited [^13^C]-glutamine flux to palmitate (via reductive flow) by 33%, and inhibited [^13^C]-glutamine flux to succinate (via oxidative flow) by 68%. Consistently, the oxidative flux to (m + 4) fumarate and malate also decreased significantly in cells treated with AGI-6780. The exact mechanism for the uneven inhibition of the reductive and oxidative fluxes by AGI-6780 is unclear, since this seems inconsistent with the thermodynamics of a competitive inhibition on an enzyme. One possible explanation could be that AGI-6780 might function as an allosteric inhibitor by binding to the interface of two IDH2 monomers as indicated by previous study^25,46^, and thus could induce IDH2 conformational changes in such a way that it differentially affects the binding of different substrates for the oxidative reaction (isocitrate and NADP+) or for the reductive reaction (α-KG, NADPH, and CO_2_). Indeed, it has been demonstrated that the binding AGI-6780 to wild-type IDH2 could induce allosteric changes that render one monomer of IDH2 in an inactive open conformation and the other monomer in a half-closed conformation, whereas, in the case of mutant IDH2, AGI-6780 could cause a conformational change that decreases the free energy of NADPH binding^46^. Thus, it seems possible that AGI-6780, as an allosteric inhibitor, could induce conformational changes of IDH2, leading to unequal inhibition of the reductive and oxidative reactions.

The increased (m + 5) α-KG and elevated cellular α-KG level in IDH2-abrogated cells is another interesting observation. Such an increase could not be simply explained by the bidirectional inhibition of the TCA metabolic flow. It seems that cancer cells could sense the suppression of the TCA cycle due to IDH2 abrogation, and enhance glutamine uptake and its flux into the TCA cycle as a compensatory response. The upregulation of PSAT1 in IDH2-knockdown cells (Fig. 2g) seems to be a possible mechanism in this compensatory adaptation, since PSAT1 is a key enzyme in the serine synthesis pathway and catalyzes the conversion of glutamate to α-KG through its aminotransferase activity (Fig. 2d). We found that PSAT1 was upregulated when IDH2 was abrogated, leading to an increase in utilization of glutamine to generate more α-KG for entry into the TCA cycle (Fig. 2d–h). Thus, the upregulation of PSAT1 contributed to the increase of α-KG when IDH2 was suppressed. These data together suggest that the active glutamine metabolism via reductive TCA metabolism might be an intrinsic metabolic property of TNBC cells, and IDH2 plays a key role in this process by converting α-KG to isocitrate.

The metabolic flow of α-KG to isocitrate catalyzed by IDH2 is particularly important for TNBC cells for at least two major reasons: generation of citrate for lipid synthesis and timely removal of α-KG to prevent degradation of HIF-1α. The citrate generated through the TCA cycle is an important metabolic intermediate for lipid synthesis. Mitochondrial citrate can be transported to cytosol, where it is broken into acetyl-CoA for the synthesis of fatty acids^15,18^. This is an important process for proliferating cancer cells to produce new lipid membranes. Our study suggests that in TNBC cells, the reductive TCA cycle seems particularly active for citrate generation using α-KG from glutamate, and IDH2 is critical for the conversion of α-KG to isocitrate in this process. Adaptation to such metabolic patterns would render TNBC cells more dependent on glutamine metabolism via the reductive TCA cycle. This is consistent with the observations by others that TNBC cells are highly dependent on glutamine for growth and consume more glutamine^47,48^. It is also important to note that α-KG is an activator of prolyl hydroxylases that catalyzes the hydroxylation of HIF-1α at two prolyl residues (P402 and P564), leading to its poly-ubiquitination by the E3 ligase von Hippel-Lindau (VHL) and degradation by proteasomes^29,49,50^. Thus, timely removal of α-KG by IDH2 in the reductive TCA flow would be critical for TNBC cells to maintain HIF-1α protein, whereas accumulation of α-KG due to IDH2 inhibition would accelerate HIF-1α degradation and subsequently compromise the ability of TNBC cells to survive under hypoxia conditions. Previous studies showed that HIF-1α was hyperactive in TNBC to promote cancer growth and metastasis^32,51,52^, but the mechanisms or factors contributing to the high basal HIF-1α in TNBC remain elusive. Our study suggests that IDH2 may play an important role in maintaining a stable level of HIF-1α by timely removal of α-KG. Indeed, specific abrogation of IDH2 by shRNA caused a significant accumulation of α-KG in TNBC cells (Fig. 2), leading to degradation of HIF-1α both in vitro and in vivo (Fig. 4). Since HIF-1α is a special transcription factor that promotes the expression of multiple glycolytic enzymes response to hypoxia, it is not surprising to see that a knockdown of IDH2 by shRNA resulted in a decreased expression of glycolytic enzymes such as LDHA and ALDOA (Fig. 4c) and a lower glycolytic activity (Fig. 3l).

The abrogation of IDH2 by shRNA or by chemical inhibitor in TNBC cells led to a significant increase in cellular α-KG, a decrease of HIF1α, and a major suppression of tumor cell growth both in vitro and in vivo, indicating an important role of IDH2 in supporting TNBC metabolism and cell proliferation. The significant impact of IDH2 abrogation on cancer cell proliferation and viability was consistently observed in multiple cell lines, including MDA-MB-231, HCC38, BT549, MCF7, and BT474 cells (Figs. 1f–I,  6a–e and Fig S2b–k), suggesting that the IDH2 might play an important role in promoting cancer cell survival in various cell models. Interestingly, when IDH2 was overexpressed in TNBC cells using forced expression vectors, we only observed a moderate decrease of cellular α-ketoglutarate associated with a modest increase in HIF1α level and a slight increase in cell proliferation (Supplementary Fig. S8g–k). A possible explanation for these observations could be that TNBC cells have already expressed a sufficient level of IDH2 to meet the needs of their metabolism and proliferation, and thus a forced expression of additional IDH2 would only have a modest effect on the already active α-KG reductive metabolism and cell proliferation. In contrast, inhibition of IDH2 in TNBC cells would be expected to cause a major disruption of α-KG reductive flow, a decrease in HIF1α, and a suppression of tumor growth.

α-KG is known to function as a co-substrate for Jumonji-C (JMJC) domain-containing histone demethylases (JHDMs) and TET demethylases, which play a major role in epigenetic regulation by altering the histone and DNA methylation and thus affect the expression of various genes^53,54^. As such, it is possible that the changes in the expression of genes involved in cell survival (Bcl2 family members) and metastasis (EMT-related molecules such as E-cad, N-cad, Snail, β-catenin) observed in the IDH2 OK cells could be the consequence of the change in cellular α-KG. It would be helpful to conduct unbiased genome-wide studies to reveal the global changes in methylation landscapes and gene expression profiles in TNBC cells with IDH2 knockdown.

Interestingly, α-KG is also known to bind the β-subunit of ATP synthase in the Complex V of the mitochondrial electron transport chain and inhibit its activity in ATP synthesis, leading to low oxygen consumption and low cellular ATP content^26^. Consistent with these observations, we showed that the elevation of α-KG due to IDH2 suppression could cause a significant decrease in OCR and in cellular ATP in TNBC cells, while direct addition of cell-permeable DM-α-KG to TNBC cell culture also caused inhibition of OCR and ATP generation (Fig. 3). Thus, the abnormal accumulation of α-KG due to in IDH2 inhibition in TNBC cells could impact two major energy metabolic pathways: a suppression of glycolysis due to α-KG-mediated degradation of HIF-1α and a reduction of mitochondrial ATP synthesis due to α-KG inhibition of Complex V. Such double-hit might explain, at least in part, why inhibition of IDH2 has such a major impact in TNBC viability. In TNBC cells, the intrinsically active glutamine anaplerotic pathway that feeds into the TCA cycle via α-KG further underscores the critical role of IDH2 in moving α-KG toward isocitrate/citrate for the synthesis of fatty acids. Thus, an active IDH2 would be very important in preventing α-KG accumulation in TNBC cells and, at the same time, promoting lipid synthesis for cell proliferation. As such, wt-IDH could be a potential therapeutic target for the treatment of TNBC.

In this study we have tested the possibility to target wild-type IDH2 as a new strategy for treatment of TNBC. In the initial proof-of-principle experiments, we showed that specific silencing of IDH2 could profoundly inhibit TNBC cell proliferation and induced apoptosis in culture (Fig. 1e–i), and could effectively suppress tumor growth in vivo (Fig. 1l). Pharmacological inhibition of IDH2 using AGI-6780 further demonstrated the feasibility of targeting this molecule for potential clinical treatment of TNBC patients. Indeed, treatment of TNBC cells with the IDH2 inhibitor AGI-6780 consistently recapitulated the key changes observed in TNBC cells with IDH2 knockdown. AGI-6780 induced a significant increase of cellular α-KG in TNBC cells (Fig. 6f, g) but not in non-cancerous MCF10A cells (Fig. 6h), and caused a major inhibition of proliferation in TNBC cells but not in MCF10A cells (Fig. 6a), suggesting that the non-cancerous MCF10A cells might be metabolically programmed very differently from that of TNBC cells, and likely do not require IDH2 to remove α-KG for survival. Importantly, AGI-6780 was able to induce ATP depletion and massive cell death in TNBC cells, and effectively suppressed tumor growth in vivo. Furthermore, the depletion of ATP compromised the ability of the ATP-dependent drug pump to export chemotherapeutic drug doxorubicin, and thus enhanced its anticancer activity in vitro and in vivo (Fig. 6l and Supplementary Fig. S11). Although AGI-6780 was initially developed as an inhibitor of mutant IDH2, this compound was shown to inhibit both mutant and wild-type IDH2^25^. Since the TNBC cell models used in this study contain wild-type IDH2, thus the observed therapeutic activity of AGI-6780 could be reasonably attributed to inhibition of wild-type IDH2. The consistent results from specific knockdown of wild-type IDH2 by shRNA further validated this conclusion.

Mutations in isocitrate dehydrogenases (IDHs) are significant oncogenic events in different cancers^55^, and targeting mutant IDHs is currently an active area of drug development. In contrast, our study demonstrated that wild-type IDH2 is essential for the survival of TNBC cells due to their special metabolic characteristics, and could be a novel target for potential clinical treatment of TNBC patients using pharmacological inhibitors.

## Methods

This research complies with all relevant ethical and safety regulations of Sun Yat-sen University Cancer Center. The animal study using research mice was performed according the protocol approved by the Institutional Animal Care and Use Committee (IACUC) of Sun Yat-sen University Cancer Center.

### Reagents and antibodies

Annexin V-FITC and propidium iodide (PI) were from BD Biosciences (San Jose, CA, USA). Octyl-α-KG was from Cayman. PX-478, AGI-6780 and Cycloheximide were from Selleckchem (Houston, TX, USA). Dimethyl-α-KG was from sigma. Antibodies to detect IDH2 (ab55271), HIF1α (ab51608), LDHA (ab47010), β-action (ab6276), BCL2 (ab32124), MCL-1 (ab32087), and ALDOA (ab169544) were from Abcam (Cambridge, UK). Antibodies to detect E-cadherin (CST3195), Snail (CST3879), β-Catenin (CST8480), and N-cadherin (CST13116) were from Cell Signaling Technology (Danvers, MA, USA).

### Cell culture

MDA-MB-231, HCC38, BT549, and MCF10A cell lines were purchased from the American Type Culture Collection (ATCC). MCF7 and BT474 cell lines were purchased from the Chinese National Infrastructure of Cell Line Resource. MDA-MB-231 was cultured in DMEM with 10% FBS; all other cells were cultured using the conditions recommended by ATCC.

### Generation of IDH2-overexpressing and IDH2-knockdown cells

The IDH2-overexpressing plasmid LV105-IDH2, empty control vector LV105, and IDH2 overexpression rescue plasmids (OV1 IDH2 rescue expression vector resistant to shRNA #1, OV2 IDH2 rescue expression vector resistant to shRNA #2) were purchased from Genecopoeia (Rockville, MD, USA); shRNA plasmids (GV-248-sh-IDH2#1 and GV-248-sh-IDH2#2) and the non-target vector GV-248 were from Genechem (Shanghai). For lentivirus production, each plasmid was co-transfected with the packaging (psPAX2) and envelope (pMD2.G) vectors into HEK293T cells. Lentivirus were harvested at 48 h post-transfection from the supernatants, and mixed with 5 mg/mL polybrene (Selleckchem) to increase the infection efficiency. MDA-MB-231, HCC38, BT549, BT474, or MCF7 breast cancer cells were infected with the lentivirus and selected in 2 ug/mL puromycin (Selleckchem) for 3 days. The shRNA and rescue sequences are shown in Supplementary Table S2.

### Cell growth, cell viability, apoptosis, and colony formation assays

Cells were seeded in a six-well plate (5 × 10^4^/well), and at the indicated time points, cell numbers were counted by Trypan blue exclusion using an auto-counting chamber. Cell viability was determined in a 96-well (2 × 10^3^) plate using the MTS reagent from Promega (Wisconsin, USA). Apoptosis was measured using an Annexin V-FITC/propidium iodide kit and a Beckman cytoFLEX flow cytometer (BD Biosciences). The flow cytometry gating strategy is shown in Supplementary Fig. S13. For colony formation, cells were seeded in six-well plates (2 × 10^3^ cells/well) and incubated for two weeks, cell colonies were stained crystal violet and counted. To determine the impact of octyl-α-KG, PX-478, or AGI-6780 on cell survival, cells were treated with different concentrations of the compounds or with solvent as a control. Then direct cell counting, MTS assay, apoptosis analysis, or colony formation were performed.

### RT-qPCR

Total RNA was isolated using the Trizol reagent (Thermo Fisher Scientific, Waltham, MA, USA), and was then converted to cDNA using a reverse transcription kit from Thermo Fisher Scientific. Quantitative RT-PCR was performed using the ABI7500 (Applied Biosystems) by mixing cDNA, primers and SYBR®Green Real-Time PCR Master Mixes (Thermo Fisher Scientific). The primer sequences are listed in Supplementary Table S3.

### Western blot analysis

Cells were harvested and washed with PBS twice, disrupted in IP buffer, and centrifuged at 12,000×*g* for 20 min. Protein (20 μg) from the supernatant fraction (quantified by the BCA Protein Assay Kit, Thermo Fisher Scientific) was subjected to SDS–PAGE, and transferred to a PVDF membrane (Millipore, Bedford, MA, USA). Membranes were blocked with 5% non-fat milk for 1 h at room temperature and then incubated with the specific primary antibody, followed by incubation with the corresponding HRP-conjugated secondary antibody. Protein bands were visualized using the Western lightening plus-ECL kit (Thermo Fisher Scientific).

### Protein stability assay

MDA-MB-231 cell was seeded into six-well plates and incubated with AGI-6780 or DMSO for 24 h. Cells were treated with CHX and collected at indicated time points for protein analysis by western blotting.

### Measurement of cellular metabolism in life cells

Oxygen consumption rate (OCR) and extracellular acidification rate (ECAR) was measured using the Seahorse XFe96 Extracellular FluxAnalyzer (Seahorse Bioscience, North Billerica, MA) according to the manufacturer’s instructions.

### Glutamine uptake analysis

Stable IDH2 knock down cells and the normal control (NC) cells (5 × 10^4^) were seeded in a six-well plate and cultured for 12 h; the culture medium was replaced with fresh medium for 24 h. Glutamine concentration was measured using YSI 2900 (YSI life sciences, Texas, USA). Glutamine uptake was calculated by glutamine consumption in the medium and was normalized by cell number.

### Metabolic flux analysis

Cells (2 × 10^6^) were seeded into 10-cm dishes and allowed to attach for 12 h. The cells were washed with PBS and the culture medium was replaced with carbon-labeled [U-^13^C] glutamine or glucose (Cambridge Isotope Laboratories) with or without treatment with AGI-6780 (Selleck) for 24 h. The cells were washed with 0.9% NaCl. Then 500 μl ice-cold methanol and 200 μl buffer containing 1 ug norvaline were added to each dish. Cells were scraped, transferred into centrifuge tubes, and were spun down at 15,000×*g* for 15 min at 4 °C. The samples was dried under vacuum for 3 h, and then subjected to GC-MS analysis as previously described^18,56,57^.

### α-KG assays

Cells (2 × 10^6^) were harvested and washed with PBS twice and centrifuged at 12,000×*g* for 10 min. α-KG was measured using an alpha-ketoglutarate colorimetric fluorometric assay kit (Biovision) according to the instructions provided by the manufacturer. Mass spectrometry analysis was also used to determine α-KG in some experiments, as indicated in the corresponding figure legends.

### Animal study

Animal experiments were conducted according to protocols approved by the Institutional Animal Care and Use Committee of Sun Yat-sen University Cancer Center. The maximal tumor size permitted by the ethics committee is 2000 mm^3^, and the tumor sizes in our study never exceed this maximal limit. Athymic nude mice (BALB/c nude, 6-week, female, from Charles River Laboratories-Beijing) were housed in the pathogen-free animal facilities of Sun Yat-sen University Cancer Center. The mice were inoculated with 2 × 10^6^ MDA-MB-231 cells, stable IDH2-overexpressing cells, IDH2-knockdown cells, or their corresponding control cells with matrigel into the fat pads. Tumor growth was recorded by measuring the length and width of the tumors. Tumor size was calculated using the formula length × width^2^/2. Tumors were harvested and weighed at the end of the experiments. For drug treatment, doxorubicin, dimethyl-α-KG, or/and AGI-6780 were injected intraperitoneally using the dose-schedules indicated in the relevant figure legends. At the end of the animal experiments, euthanasia of the mice was performed using cervical dislocation. Tumors were isolated, weighed, and processed for pathological and immunohistochemistry analyses as indicated.

### Lung metastasis model

MDA-MB-231 cells (1 × 10^6^ cells/mouse) in 100 μl PBS were injected into the mouse tail vein. After 35 days, lung tissues were collected for histological analysis as previously described^19^.

### Statistics and reproducibility

The statistical differences in tumor areas in the lungs of mice injected (via tail vein) with TNBC cells with different IDH2 expression status were evaluated by a paired student’s *t*-test. The statistical differences in tumor growth among MDA-MB-231 xenografts under different experimental conditions were analyzed using a two-way ANOVA test. Other statistical analyses of in vitro experimental data are indicated under the respective figure legends. Two-tailed unpaired *t*-tests, two-way ANOVA test, and correlation analysis were conducted using the software provided with GraphPad Prism 7. A *p* value of less than 0.05 was considered statistically significant.

No statistical method was used to predetermine the sample size. Animals were randomly assigned to different experimental groups, and the Investigators were not blinded to the allocation during experiments and outcome assessment. No data were excluded from the analyses.

### Reporting summary

Further information on research design is available in the Nature Portfolio Reporting Summary linked to this article.

### Supplementary information

Supplementary Information

Description of Additional Supplementary Files

Supplementary Data 1

Supplementary Data 2

Supplementary Data 3

Supplementary Data 4

Reporting Summary

### Source data

Source Data

## Data Availability

All the data in this study are included in the published article files. Source data are provided with this paper. Datasets available in the public databases, including cBioprotal (TCGA) and Oncomine, were used to analyze the potential relationship between genomic changes and mRNA expression in cancers as described previously^58–60^. Kmplot was used to compare the survival of cancer patients with different levels of IDH2 expression. Publicly available datasets used in our analyses were from TCGA and NCBI (GEO) databases, including GSE11121, GSE12093, GSE12276, GSE1456, GSE16391, GSE16446, GSE16716, GSE17705, GSE17907, GSE18728, GSE19615, GSE20194, GSE20271, GSE2034, GES20685, GSE20711, GSE21653, GSE22093, GSE25066, GSE2603, GSE26971, GSE29044, GSE2990, GSE31448, GSE31519, GSE32646, GSE3494, GSE36771, GSE37946, GSE41998, GSE42568, GSE43358, GSE43365, GSE45255, GSE4644, GSE46184, GSE48390, GSE50948, GSE5327, GSE58812, GSE61304, GSE65194, GSE6532, GSE69031, GSE7390, GSE76275, GSE78958, and GSE9195^61^. Survexpress was utilized to analyze IDH2 mRNA expression in breast cancer tissues with different pathological types and clinical stages, using the TCGA datasets^62^. The Cancer Cell Line Encyclopedia (CCLE) was used to analyze the correlation between gene copy numbers and mRNA expression. Source data are provided with this paper.
