# Supplementary material for: Wild-type IDH2 is a therapeutic target for triple-negative breast cancer
Source: Nat Commun. 2024 Apr 24;15:3445. doi: 10.1038/s41467-024-47536-6 (PMC11043430; doi:10.1038/s41467-024-47536-6)
Supplement: Supplementary file 1 — Supplementary Information [file 41467_2024_47536_MOESM1_ESM.pdf]

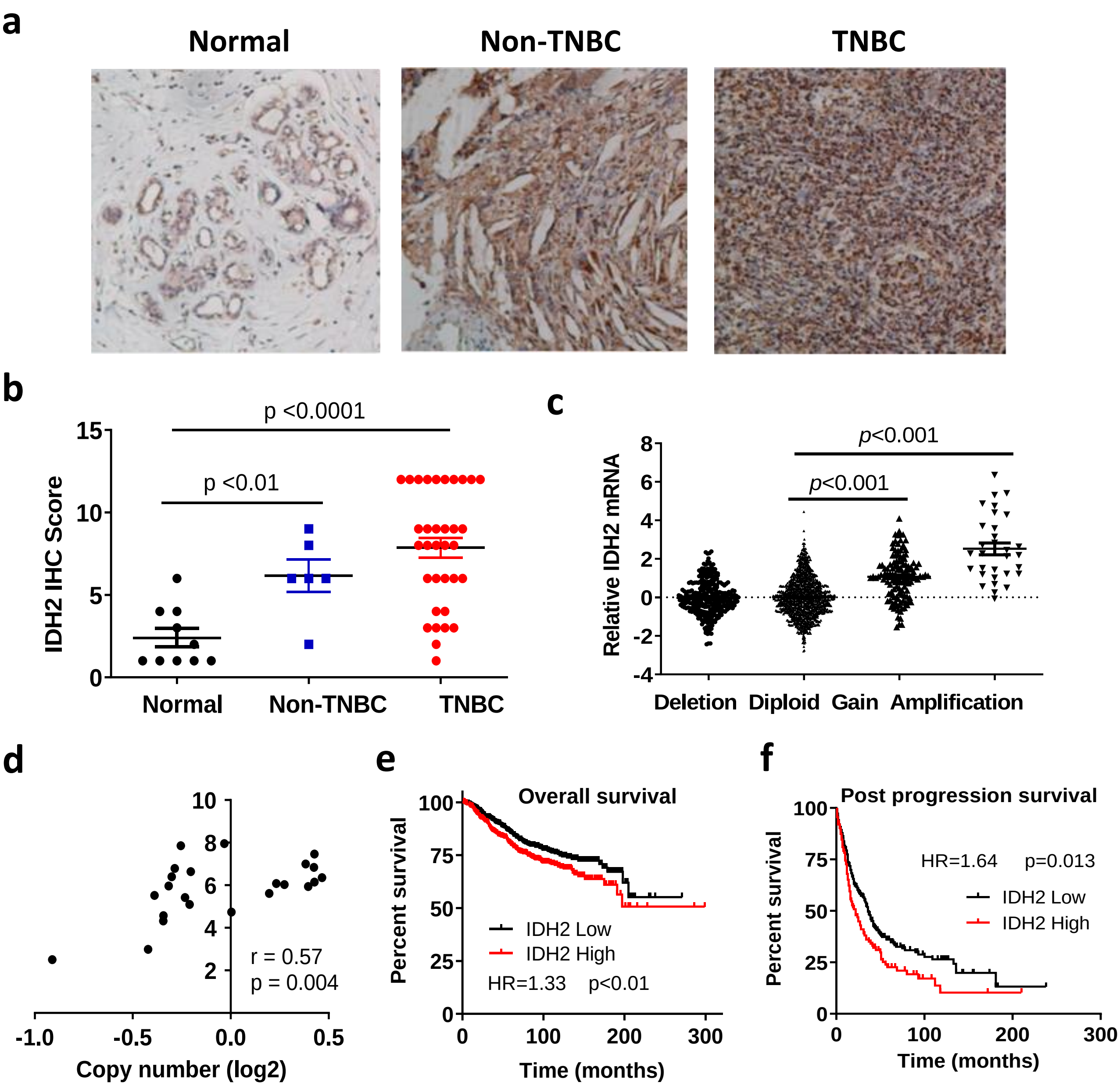

Supplementary Fig. S1. Analysis of IDH2 expression in breast cancer tissues and its association with patient survival. **a, b** Immunohistochemistry analysis of IDH2 protein in breast cancer and the adjacent normal breast tissues (n=10 for normal, n=6 for non-TNBC and n=34 for TNBC). **c** Expression of IDH2 mRNA in breast cancer samples with various genetic alterations, n=311 for deletion, n=1430 for diploid, n=130 for gain and n=31 for amplification (analysis of the genome sequencing datasets from The Cancer Genome Atlas). **d** Relationship between IDH2 mRNA expression and gene copy numbers in TNBC cell lines, n=23 (analysis of CCLE datasets). **e, f** High expression of IDH2 mRNA in breast cancer was associated with poor overall patient survival, n=701 for each group (e) and poor post progression survival, n=297 patients for the low expression group and n=117 patients for the high expression group (f). The median mRNA value was used as the cut off point (analysis of datasets from KmPlot).

Supplementary Fig. S2 (page 1)

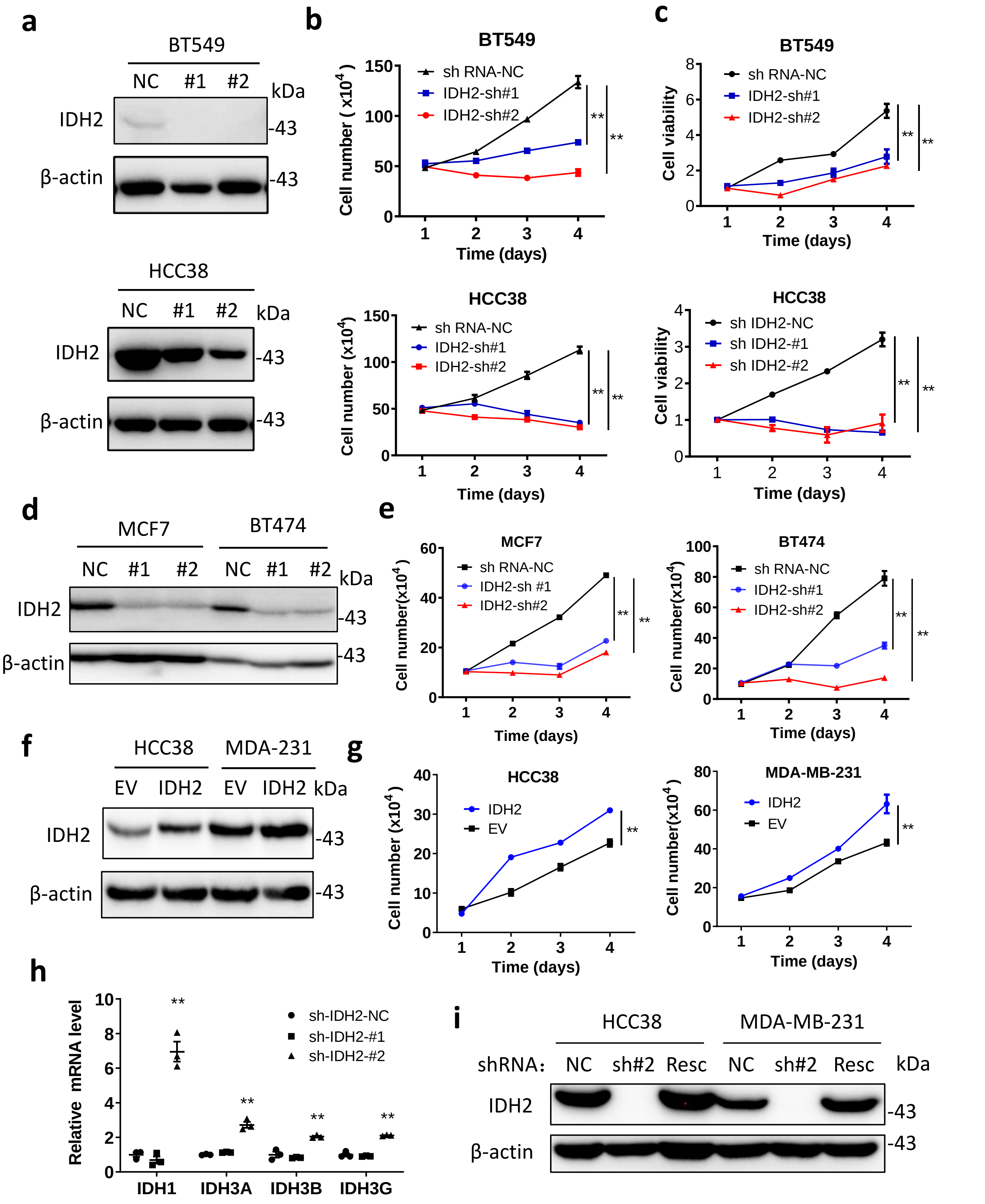

Supplementary Fig. S2 (page 2)

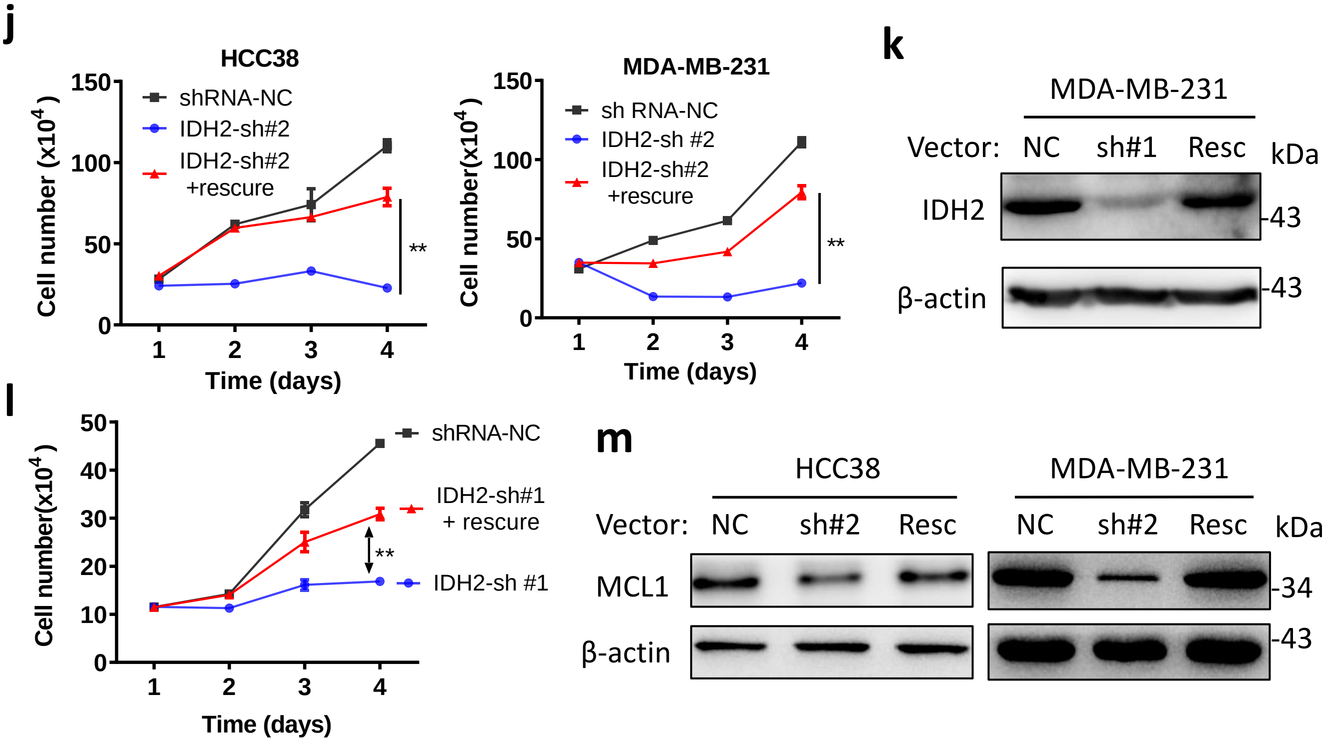

Supplementary Fig. S2. The impact of IDH2 knockdown by shRNA on TNBC cell proliferation and survival. **a** Western blot analysis of IDH2 in BT549 and HCC38 cells stably transfected with IDH2 shRNA (#1 & #2) or non-targeting control RNA (NC). The samples derive from the same experiment but different gels for IDH2 and β-actin were processed in parallel. **b-e** Effect of IDH2 silencing by shRNA on cell proliferation measured by cell number (b,e); cell viability was measured by MTS assay (c) in breast cancer cells. Note: TNBC cells: BT549 and HCC38; non-TNBC cells: MCF7 and BT474, n=3 (technical replicates) for each group. IDH2 knockdown was validated by Western blotting with β-actin run on the same gel as a loading control. (d). **f, g** Effect of IDH2 overexpression on cell proliferation measured by counting cell numbers. For Western blot analysis in (f), the samples derive from the same experiment but different gels for IDH2 and β-actin were processed in parallel. **h** The mRNA level of IDH isoenzymes in TNBC cells transfected with IDH2 shRNA (#1 & #2) or non-targeting control RNA (n=3, technical replicates) for each group. **i, j** Western blot (i) and cell number analysis of HCC38 and MDA-MB-231 cells stably transfected with IDH2 shRNA #2, non-targeting control RNA (NC) or IDH2 shRNA #2 plus IHD2 rescue expression vector resistant to shRNA #2 (n=3, technical replicates). For Western blot analysis in (i), the samples derive from the same experiment but different gels for IDH2 and β-actin were processed in parallel. **k, l** Western blot(k) and cell number analysis of MDA-MB-231 cells stably transfected with IDH2 shRNA #1, non-targeting control RNA (NC) or IDH2 shRNA #1 plus IHD2 rescue expression vector resistant to shRNA #1 (n=3 for each group, technical replicates). For Western blot analysis in (k), the samples derive from the same experiment but different gels for IDH2 and β-actin were processed in parallel. **m** Western blot analysis of MCL1 in HCC38 and MDA-MB-231 cells stably transfected with non-targeting control RNA (NC), IDH2 shRNA #2, or IDH2 shRNA #2 plus IDH2 rescue expression vector resistant to shRNA #2. β-actin was analyzed in the same gel as a loading control.

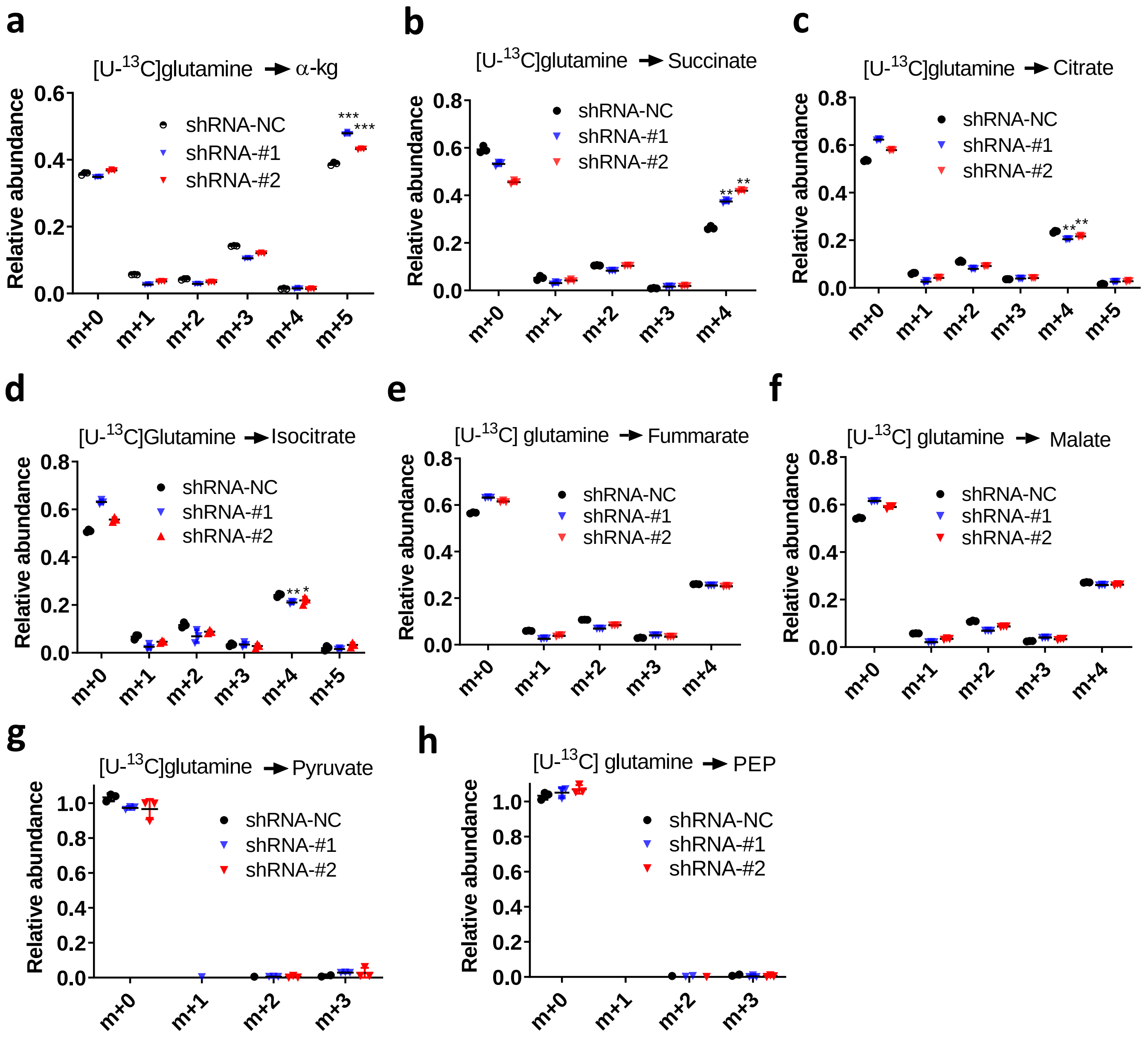

Supplementary Fig. S3. Glutamine metabolic flux analysis in TNBC cells with or without IDH2 abrogation. **a-f** MDA-MB-231 cells stably transfected with IDH2 shRNA (#1, #2) or with non-targeting control shRNA (shRNA-NC) were labeled with [U-^{13}C]-glutamine. The indicated TCA cycle metabolites in the cell extracts were analyzed by GC-MS, n=3 for each group (technical replicates). **g, h** The indicated metabolites pyruvate and phosphoenolpyruvate (PEP) in the cell extracts of MDA-MB-231 cells with stable transfection of IDH2 shRNA (#1, #2) or non-targeting control (shRNA-NC) were analyzed by GC-MS, n=3 (technical replicates) for each conditions.

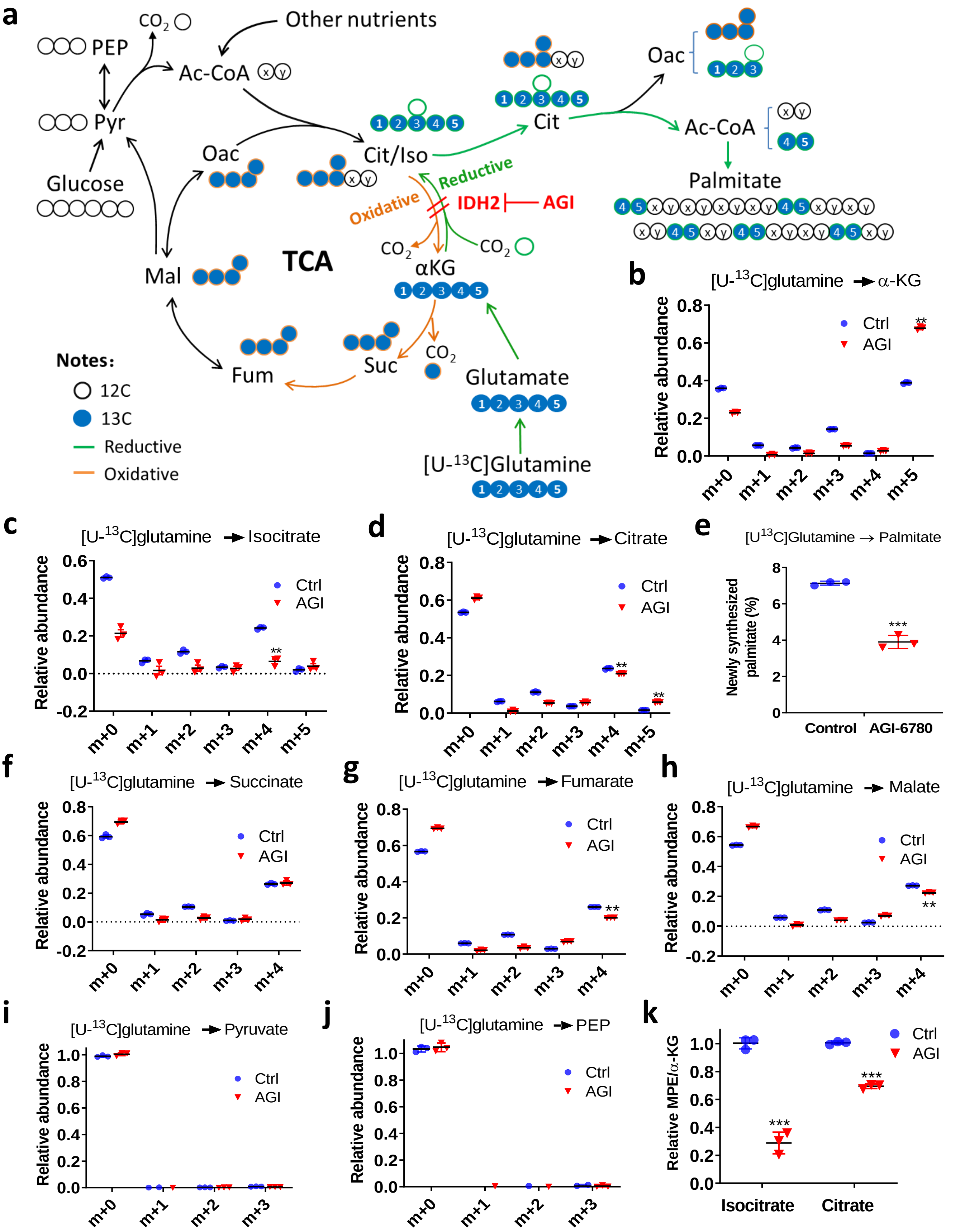

Supplementary Fig. S4. Glutamine metabolic flux analysis in TNBC cells with or without IDH2 inhibition. **a** Schematic illustration of the TCA cycle and [U^{13}C]-glutamine flux to

α-KG and the subsequent TCA metabolites and *de novo* fatty acid synthesis. The carbon sources from unlabeled glucose and other nutrients are also shown. The green lines/circles indicate [U^{13}C]-glutamine flux to palmitate via reductive TCA metabolism, while the orange lines/circles indicate oxidative TCA flow. Note that the metabolic flow from [U^{13}C]-glutamine to (M+2) acetyl-CoA (Ac-CoA) occurs mainly via reductive TCA metabolism, whereas the (M+4) citrate is from oxidative TCA product (M+4) oxaloacetate (Oac) and the unlabeled Ac-CoA; **b-j** MDA-MB-231 cells treated with IDH2 inhibitor AGI-6780 or with solvent control (DMSO) were labeled with [U-^{13}C]-glutamine, and the indicated metabolites were analyzed by GC-MS. **k** Relative levels of isocitrate/citrate, expressed as mole percent enrichment (MPE) normalized by a-KG; PEP: phosphoenolpyruvate. Student's t test was used for statistical analysis. Error bars represent mean values $\pm$ S.D. n=3 (technical replicates) for each group; **, $p \leq 0.01$; ***, ≤ 0.001. Data are representative of two independent experiments.

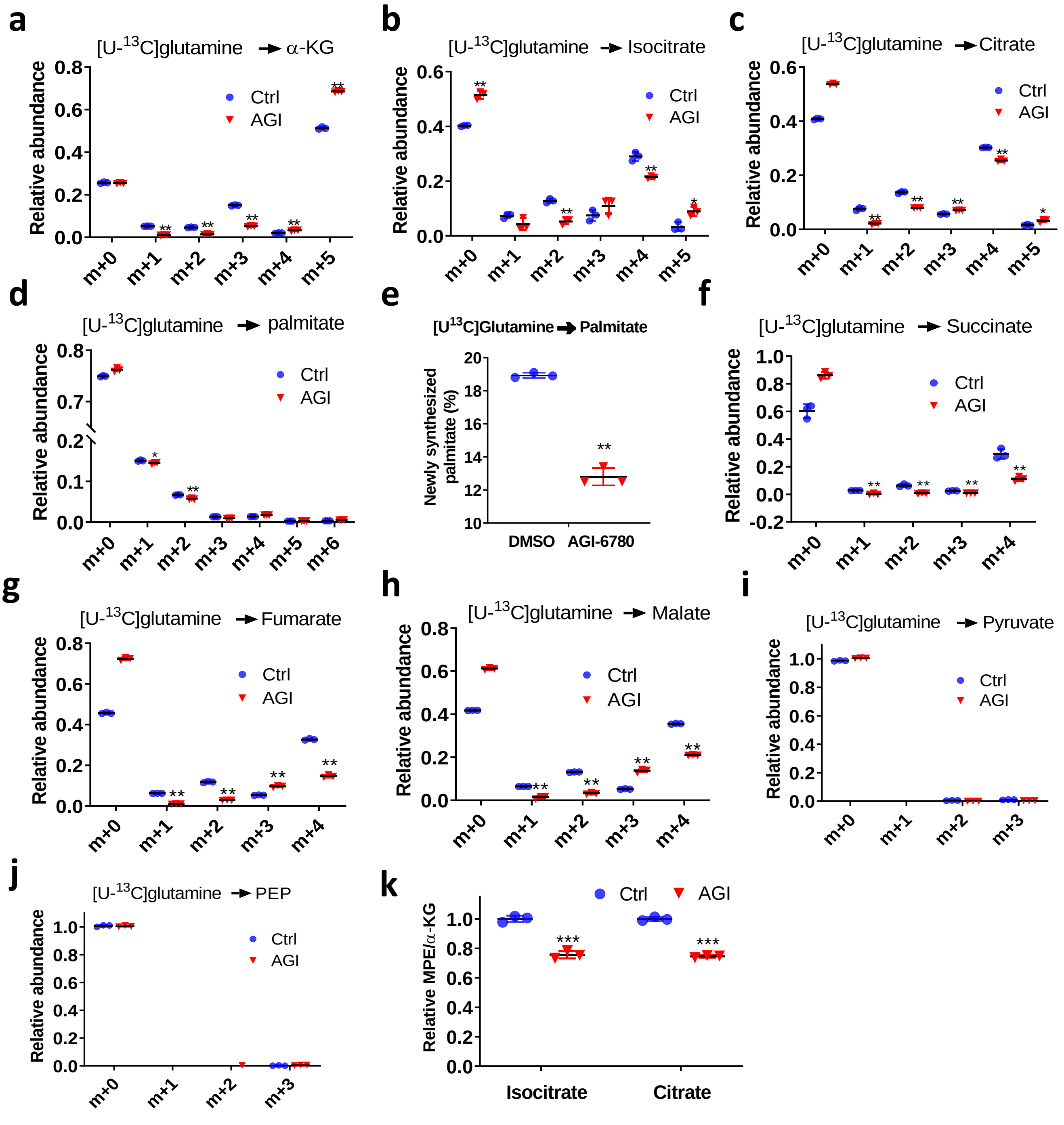

Supplementary Fig. S5. Glutamine metabolic flux analysis in TNBC cells. **a-j** MDA-MB-231 cells treated with IDH2 inhibitor AGI-6780 or with solvent control (DMSO) were labeled with [U-^{13}C]-glutamine; the indicated metabolites were analyzed by GC-MS. The MID data for each metabolite are provided in *Supplemental data file #*1. The MID data are shown in *Supplemental data file #*2. The effect of AGI-6780 on the relative abundance of the newly synthesized palmitate (panel e) was calculated using the MID data provided in *Supplemental data file #3*. **k** Relative levels of isocitrate and citrate is expressed as mole percent enrichment (MPE) normalized by α-KG. Student's t test was used for statistical analysis. Error bars represent mean values ± S.D. n=3 (technical replicates) for each group; data are representative of two independent experiments. **, p≤0.01; ***, ≤0.001.

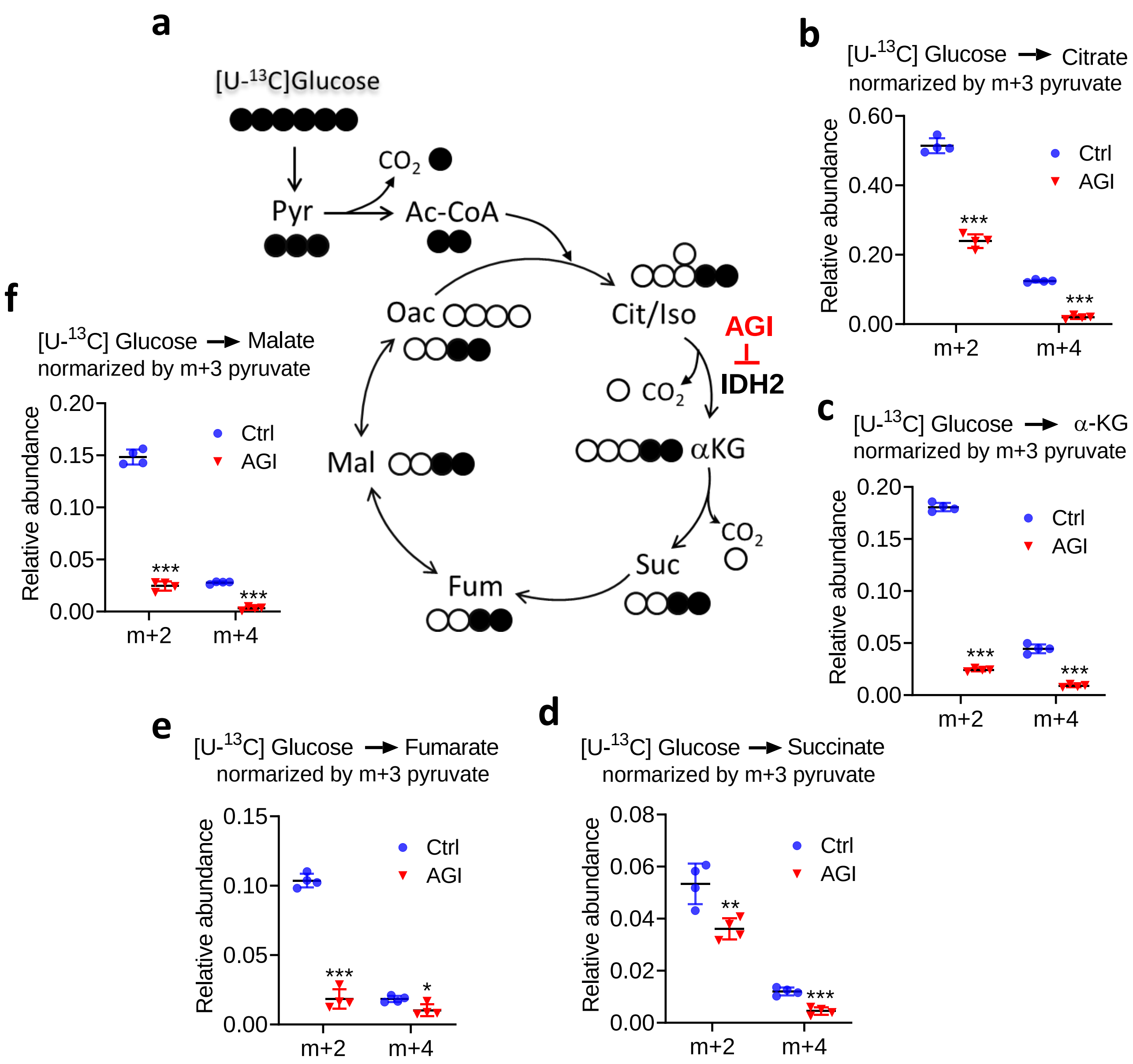

Supplementary Fig. S6. Glucose metabolic flux analysis in TNBC cells. **a** Schematic illustration of [U13C]-glucose flux through pyruvate to the TCA cycle, with the first round of [^{13}C]-labeled metabolites indicated by the dark circles. **b-f,** MDA-MB-231 cells treated with IDH2 inhibitor AGI-6780 (30 μM) or with solvent control (DMSO) were labeled with [U-^{13}C]-glucose for 24 h, and the indicated metabolites were analyzed by GC-MS. Each metabolite of the TCA cycle was normalized by (M+3) pyruvate, and the normalized labeled metabolites (m+2 and m+4) were calculated to show the relative activity of the oxidative TCA flux from (m+3) pyruvate. Student's t test was used for statistical analysis. Error bars represent mean values ± S.D. n=4 for each group (technical replicates). **, p<0.01; ***, p<0.001.

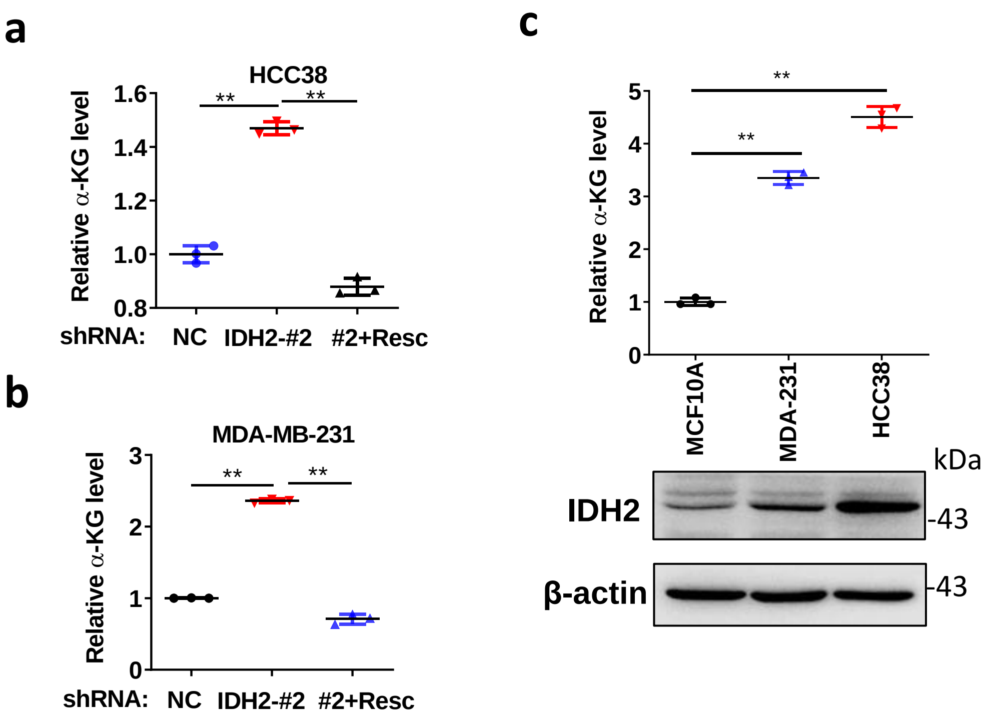

Supplementary Fig. S7. Effect of IDH2 abrogation on cellular α-KG in TNBC cells with or without and rescue. **a, b** TNBC cells (HCC38, MDA-MB-231) were stably transfected with non-targeting control RNA (NC), IDH2 shRNA #2, or IDH2 shRNA #2 plus IDH2 rescue expression vector resistant to shRNA #2. Cellular α-KG levels were then measured, n=3 for each group (technical replicates). **c** Intracellular α-ketoglutarate (n=3, technical replicates) in TNBC cell lines (MDA-231, HCC38) and normal breast cell line (MCF10A). Their expression of IDH2 was measured by western blot analysis. The samples derive from the same experiment but different gels for IDH2 and β-actin were processed in parallel.

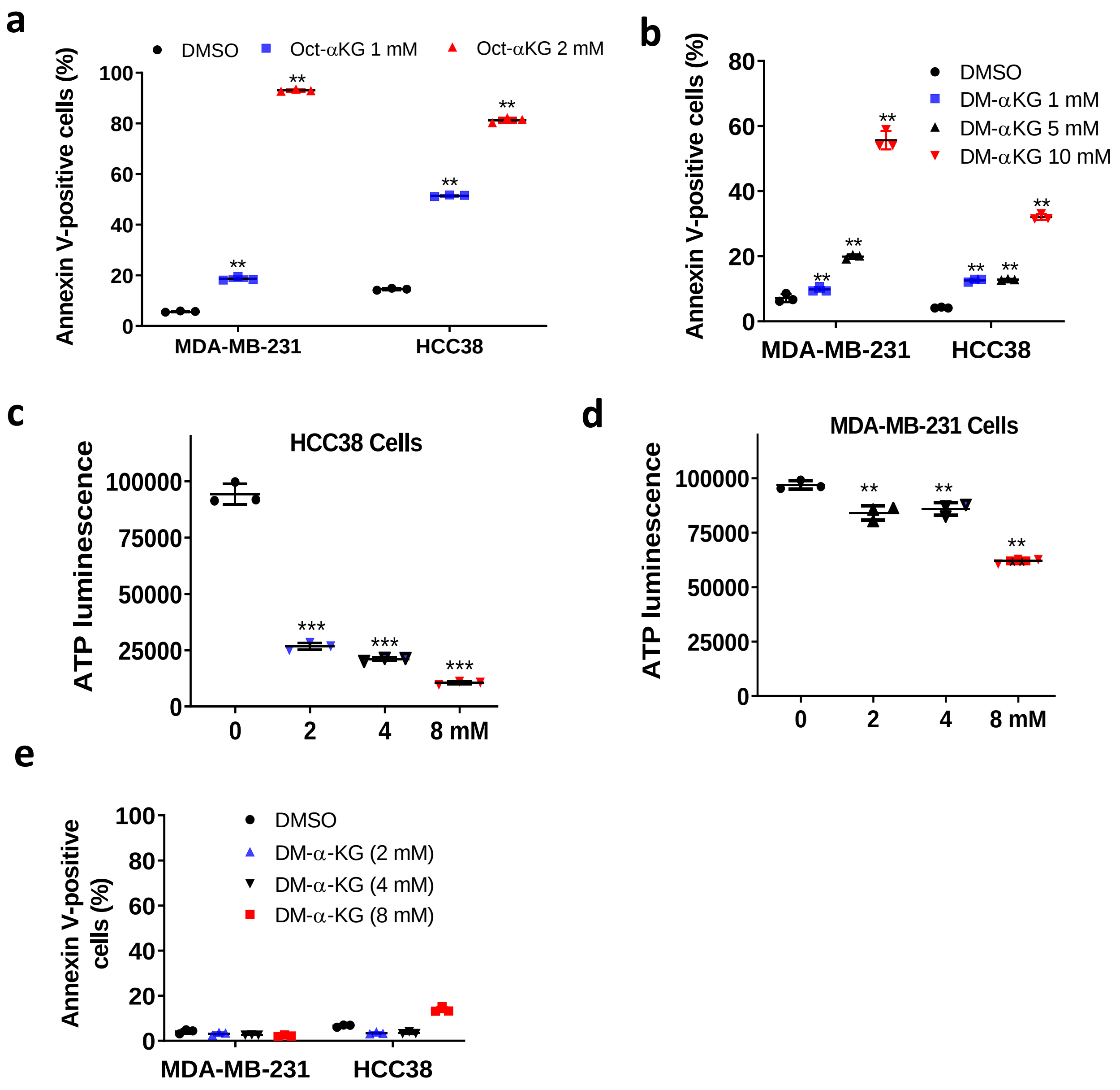

Supplementary Fig. S8. Induction of apoptosis and deplete intracellular ATP by cell-permeable α-KG in TNBC cells. **a, b** MDA-MB-231 or HCC38 cells were treated with (a) Oct-α-KG or (b) DM-α-KG for 48 h. The cells were then stained with Annexin V and PI for Flow cytometry analysis. **c, d** HCC38 and MDA-MB-231 cells were treated with DM-α-KG for 12 h, and cellular ATP was measured using a ATP luminescence kit. **e** MDA-MB-231 or HCC38 cells were treated with DM-α-KG for 12 hours. The cells were then stained with Annexin V, and quantified by Flow cytometry analysis. All experiments were performed in triplicates (n=3, technical replicates).

Supplementary Fig. S9 (page 1)

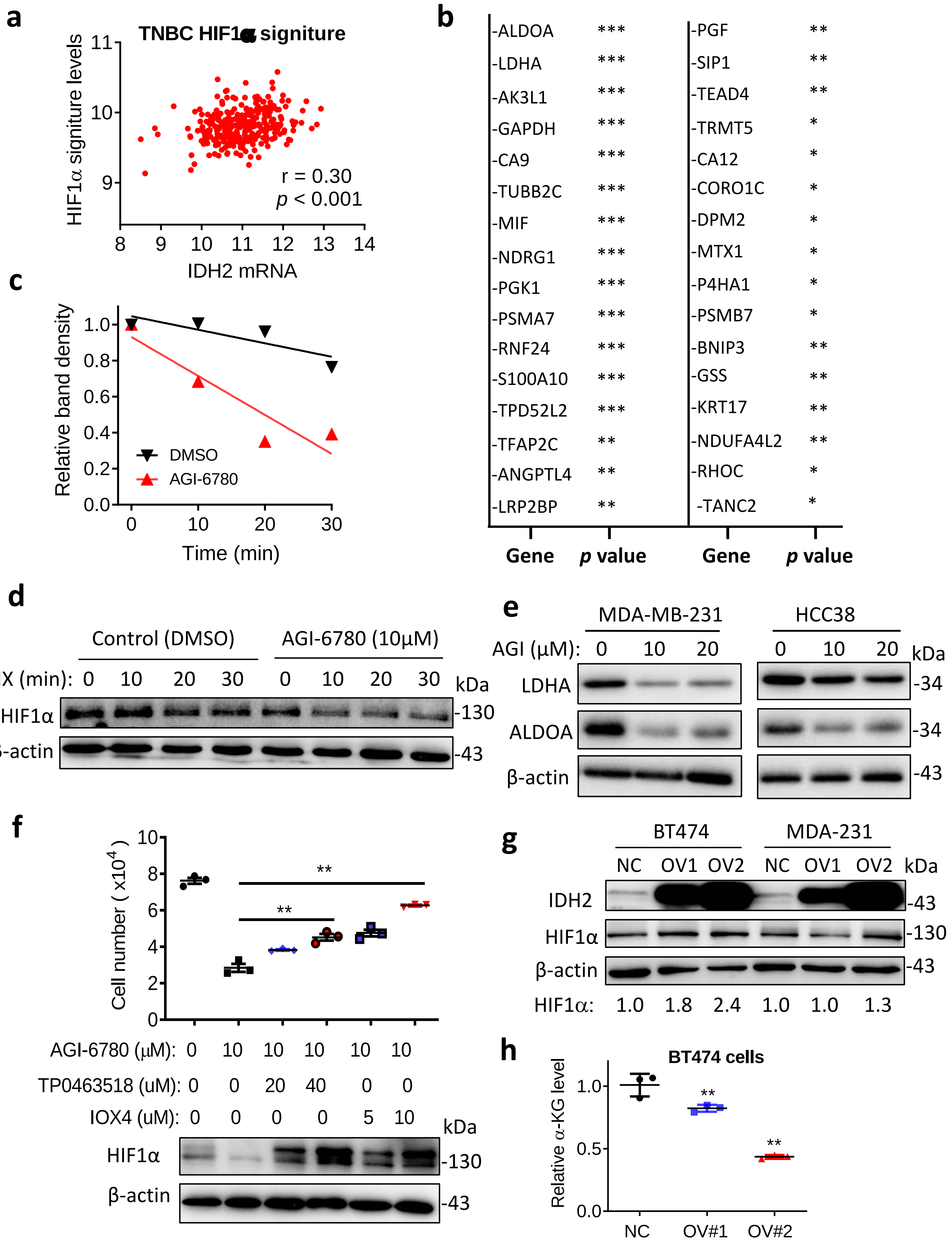

Supplementary Fig. S9 (page 2)

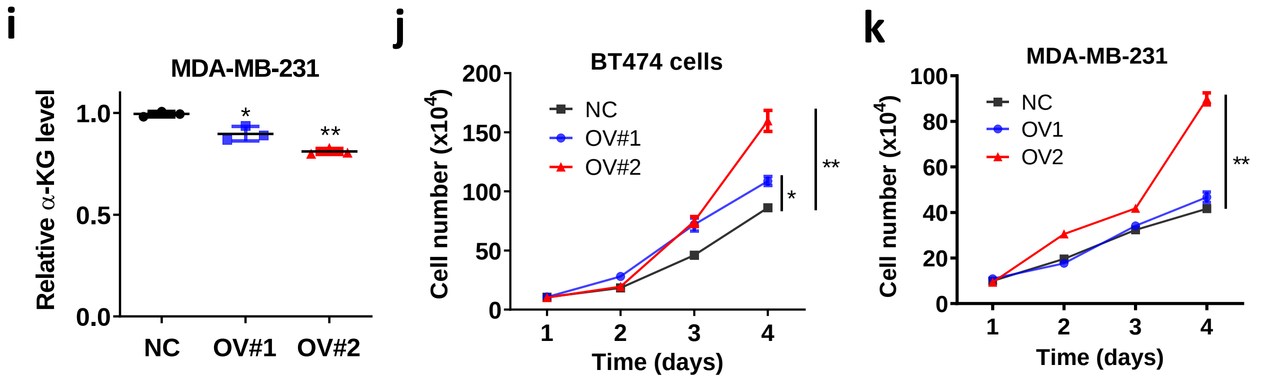

Supplementary Figure S9. Effect of IDH2 on the expression of HIF1α-related molecules in TNBC cells. **a, b** Pearson analysis of the relationship between IDH2 expression and the expression of the HIF1α-signature genes (a) or between IDH2 expression and the expression of the indicated HIF1α- target genes (b) in TNBC patient samples (n=315, dataset from Survexpress). *, p≤0.05; **, p≤0.01; ***, p≤0.001. **c, d** Impact of IDH2 inhibition on HIF1α protein stability. TNBC cells were pretreated with AGI-6780 for 24 hours, and then subjected to incubation with cycloheximide (CHX, 20 uM) for the indicated time (0-30 min). HIF-1α in the cell lysates was analyzed by western blot analysis with β-actin analyzed in the same gel as a loading control (d). Each HIF-1α band density was normalized by the corresponding β-actin band run in the same gel, and plotted as the function of time to show the protein degradation slops (c). **e** Western blot analysis of HIF1α-target genes (LDHA, ALDOS) in TNBC cells in the presence of IDH2 inhibitor AGI-6780. The samples derive from the same experiment but different gels (one gel for ALDOS and β-actin, another gel for ALDOA) were processed in parallel. **f** HCC38 cells were pretreated with PHD inhibitors TP0463518 or IOX4 for 6 hours and then incubated with IDH2 inhibitor AGI-6780 for 48 hours, and cell number and HIF-1α protein were measured. β-actin was analyzed in the same gel as a loading control. **g** Western blot analysis of HIF-1α and IDH2 expression in BT474 or MDA-MB-231 cells stably transfected with non-targeting sequence or IDH2 over-expression sequences (OV1: same plasmid as IDH2 rescue expression vector resistant to shRNA #1, OV2: same plasmid as IDH2 rescue expression vector resistant to shRNA #2), HIF-1α band density was normalized by the corresponding β-actin band run in the same gel; IDH2 was run in parallel in a separate gel. **h, i** BT474 or MDA-MB-231 cells were stably transfected with non-targeting control RNA (NC), IDH2 rescue expression vector resistant to shRNA #1 (OV1) or IDH2 rescue vector resistant to shRNA #2 (OV2). Cellular a-KG levels were then measured, n=3 (technical replicates) for each group. **j, k** Cell growth curves of BT474 and MDA-MB-231 cells stably transfected with non-targeting control RNA (NC), IDH2 overexpression vectors OV1 or OV2 in different time points, n=3 (technical replicates) for each group.

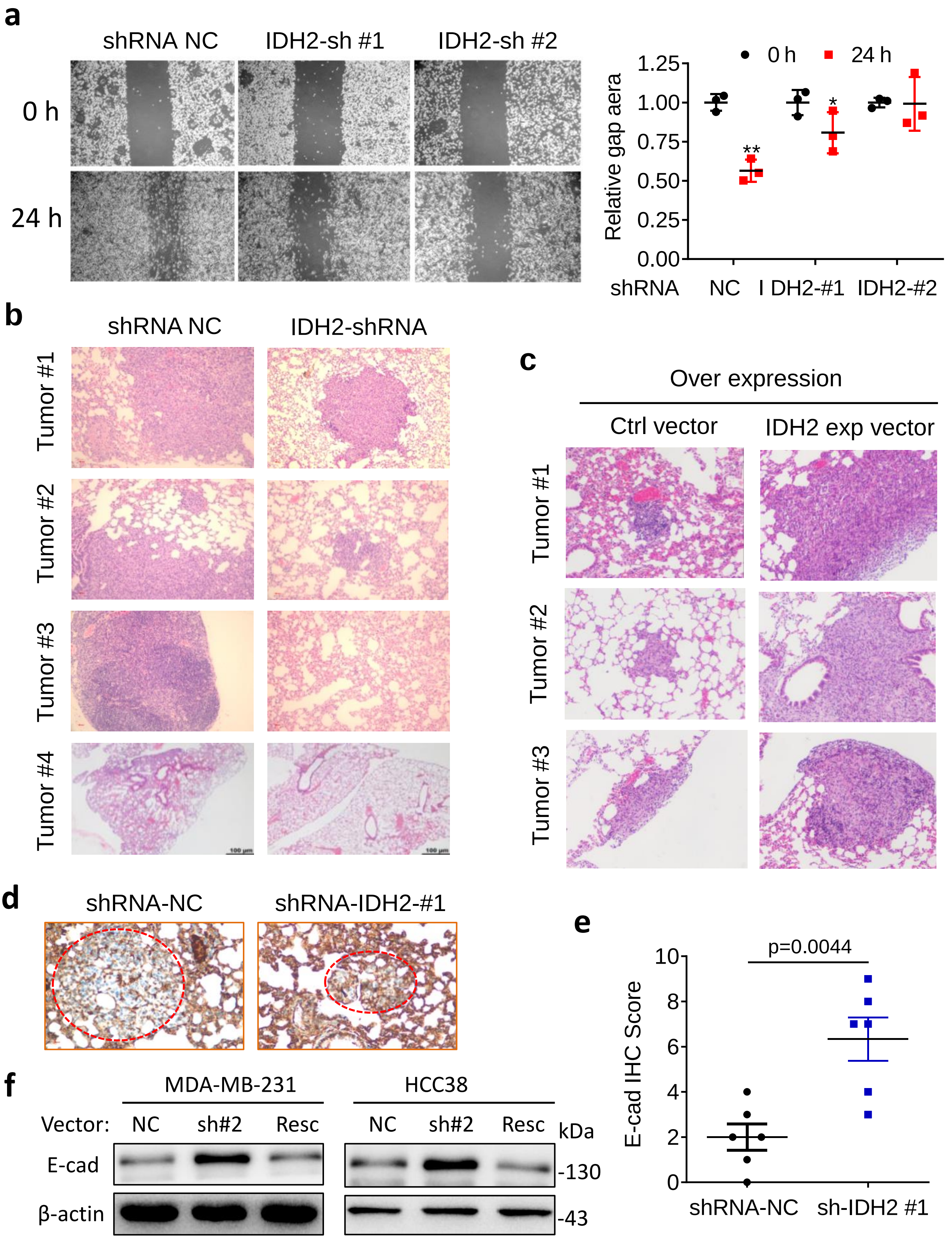

Supplementary Fig. S10. Effect of IDH2 knockdown on TNBC cell migration and metastasis. **a** MDA-MB-231 cells were stably transfected with IDH2 shRNA (#1 or #2) or

with non-targeting control shRNA, and the effect of IDH2 knockdown on cell migration was evaluated by wound healing assay. Images were photographed immediately after the cells were scratched (0 h) and at 24 hours. The cell gap areas were quantified using the Image J software; n=3 (technical replicates) for each group. Data are representative of two independent experiments. Student's t test was used for statistical analysis. Error bars represent mean values ± S.D.; *, $p \le 0.05$; **, ≤0.01. **b, c** Hematoxylin-Eosin (HE) staining of the lung tissues from nude mice injected (via tail veins) with of MDA-MB-231 cells transfected with IDH2 shRNA (b) or with non-targeting control RNA (NC), or with IDH2-overexpression vector (c). **d, e** IHC staining of the E-cadherin protein (E-cad) in lung tissues from nude mice injected (via tail veins) with MDA-MB-231 cells transfected with IDH2 shRNA-#1 or with non-targeting control RNA (NC). The red dashed circles in (d) indicate the lung metastatic tumor foci; n=6 tumors for each group in (e). **f** Western blot analysis of E-cadherin in MDA-MB-231 or HCC38 cells stably transfected with non-targeting control RNA (NC), IDH2 shRNA #2, or IDH2 shRNA #2 plus IDH2 rescue expression vector resistant to shRNA #2. β-actin was analyzed in the same gel as the loading control.

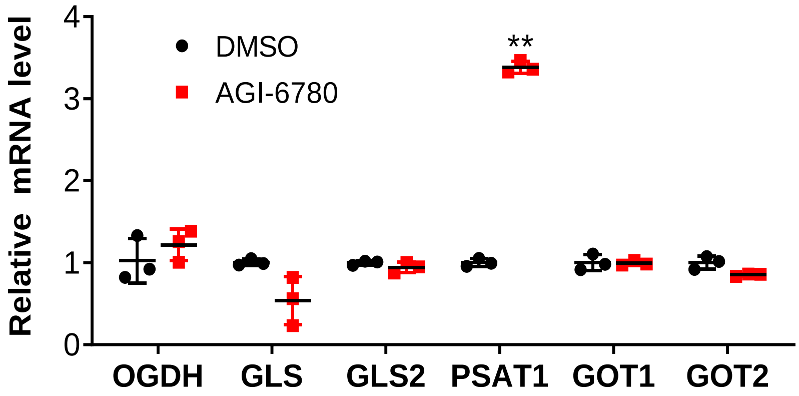

Supplementary Fig. S11. Effect of AGI-6780 on the expression of the enzymes involved in glutamine and α-KG metabolism. MDA-MB-231 cells were treated with AGI-6780 (10 uM) or the solvent control (diluted DMSO) for 48 hours. Cellular RNA was isolated and the expression of the indicated genes was measured by qRT-PCR. Student's t test was used for statistical analysis. Error bars represent mean values ± S.D., n=3 (technical replicates) for each group; **, p≤0.01.

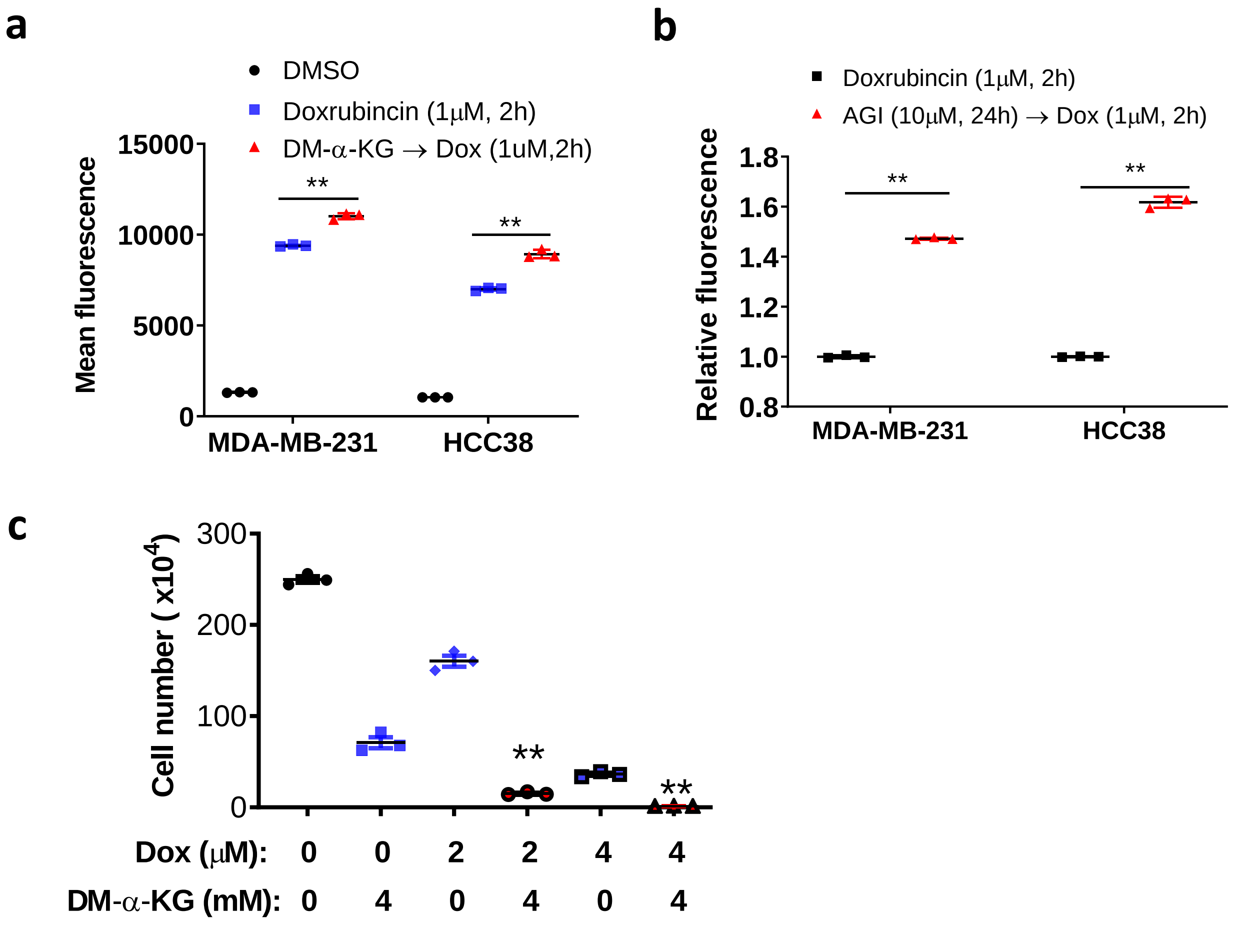

Supplementary Fig. S12. Potentiation of doxorubicin effect against TNBC cells by cell permeable DM-α-KG. **a** MDA-MB-231 or HCC38 cells were treated with doxrubincin (1 μM, 2 h), or with DM-α-KG (4 mM, 12 h) and then with doxrubincin (1 μM, 2 h) as indicated. Intracellular doxrubincin contents were measured by detection of the intrinsic fluorescence of doxorubicin using flow cytometer analysis. **b** MDA-MB-231 or HCC38 cells were treated with doxrubincin (1 μM, 2 h), or with AGI-6780 (10 uM, 24h) and then with doxrubincin (1 μM, 2 h) as indicated. Intracellular doxrubincin contents were measured by detection of the intrinsic fluorescence of doxorubicin using flow cytometer analysis. **c** HCC-38 cells treated with the indicated concentrations of with doxrubincin, DM-α-KG, or their combination for 48 hours, viable cells were quantified by Trypan blue exclusion assay; n=3 (technical replicates) for each group. Student's t test was used for statistical analysis. Error bars represent mean values ± S.D.; **, p≤0.01.

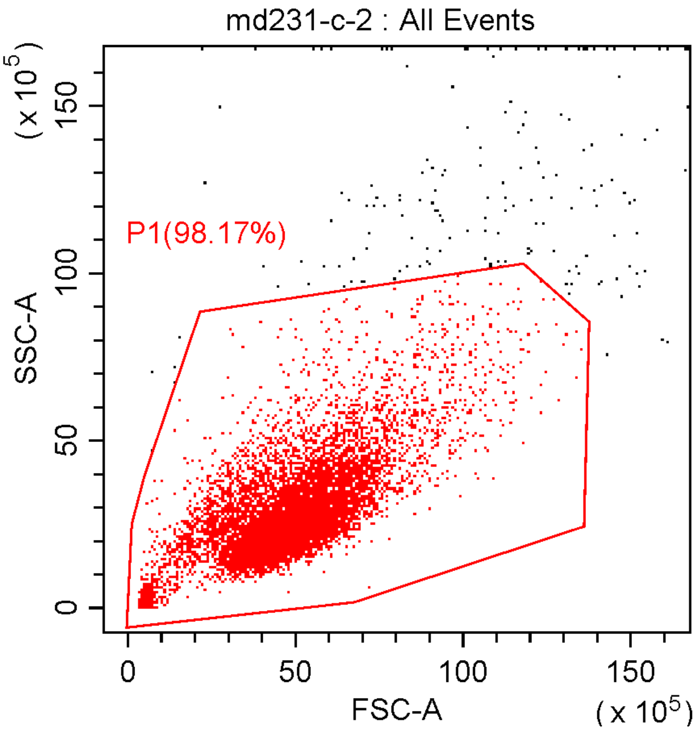

Supplementary Fig. S13 Flow cytometry gating method for apoptosis assay.

Supplementary Table S1. IDH2 expression in breast cancer tissues and normal tissues.

Datasets	Cancer vs. Normal	
	**Fold** (Cancer/Normal)	**p value**
Radvanyi (GEO)<br>Invasive ductal breast carcinoma	2.71	5.30E-4
Curtis (GEO)<br>Invasive ductal breast carcinoma	2.53	2.44E-80
Ma (GEO)<br>Ductal breast carcinoma	1.93	1.13E-4
Gluck (GEO)<br>Invasive ductal breast carcinoma	2.15	2.00E-3
Karnoub (GEO)<br>Invasive Ductal BreastCarcinoma	2.01	1.00E-3
Invasive Ductal Breast (TCGA)	2.89	6.23E-33
Mixed Lobular and Ductal Breast Carcinoma (TCGA)	2.24	2.17E-5
Invasive Breast Carcinoma (TCGA)	2.45	2.38E-20
Invasive Lobular Breast Carcinoma (TCGA)	1.87	1.02E-8
Invasive Ductal and Lobular Carcinoma (TCGA)	3.56	3.00E-3

The mRNA level of IDH2 in cancer and normal tissues was compared and analyzed using the original datasets from GEO and TCGA database and the analytical tools from Oncomine website. Two-sided Student's t test was used for statistical analysis.

Supplementary Table S2. Target sequences of the IDH2 shRNA and rescue plasmids.

Plasmids	Target Sequences (5'-3')
shRNA-NC	TTCTCCGAACGTGTCACGT
IDH2-#1	TGTACATGAGCACCAAGAA
IDH2-#2	GGACATCCAGCTAAAGTAT
IDH2-#1-rescue	TCTATATGTCTACTAAAAA
IDH2-#2-rescue	CGATATACAACTCAAATAC

Supplementary Table S3. Primer sequences used for qRT-PCR.

Gene	Forward	Reverse
ALDOA	GCCCGTTATGCCAGTATCT	AGCCAAGACCTTCTCTGTAA
LDHA	GGTTGAGAGTGCTTATGA	AACACTAAGGAAGACATCA
OGDH	TGGTAGAAGCACAGCCCAAC	AGGCCATAGAACCCAAGTTTGT
GLS	AAGGAATGCCTTTGATCACCAC	AGGATCAGTGGCAGCCTACA
GLS2	AGGATCAGTGGCAGCCTACA	TGGGGTTATGGGGGATTCCT
PSAT1	TGCCGCACTCAGTGTTGTTA	GCTAGCAATTCCCGCACAAG
GOT1	AGAAGCCCTCAAAACCCCTG	CGTTGATTCGACCACTTGGC
GOT2	ACCCATGTGGAAATGGGACC	GACTTCGCTGTTCTCACCCA
IDH1	CACCAAATGGCACCATACGAA	CCCCATAAGCATGACGACCTAT
IDH3A	TGCTGCCAAAGCACCTATTCA	GTGACCGGCTGCTATTGGG
IDH3B	TAGTGCAGAATACGCAGTCTTTG	CAGCATGTTGGAAGCCGAC
IDH3G	AAACAATTCCTCCGTCCGCTA	ATGGCATTGCGAATGTCCTCT
