# Supplementary material for: Wild-type IDH2 is a therapeutic target for triple-negative breast cancer
Source: Nat Commun. 2024 Apr 24;15:3445. doi: 10.1038/s41467-024-47536-6 (PMC11043430; doi:10.1038/s41467-024-47536-6)
Supplement: Supplementary file 2 — Description of Additional Supplementary Files [file 41467_2024_47536_MOESM2_ESM.pdf]

Description of Additional Supplementary Files

File Name: Supplementary Data 1
Description: file containing MID data of[C13]Glutamine flux experiment.

File Name: Supplementary Data 2
Description: file containing MID data for calculating (iso)citrate MPE.

File Name: Supplementary Data 3
Description: file containing MID data for calculating newly synthesized Palmitate.

File Name: Supplementary Data 4
Description: file containing [C13]Glucose flux MID data normalize by m+3 pyruvate.
