# Supplementary material for: Wild-type IDH2 is a therapeutic target for triple-negative breast cancer
Source: Nat Commun. 2024 Apr 24;15:3445. doi: 10.1038/s41467-024-47536-6 (PMC11043430; doi:10.1038/s41467-024-47536-6)
Supplement: Supplementary file 7 — Reporting Summary [file 41467_2024_47536_MOESM7_ESM.pdf]

Corresponding author(s): Peng Huang
Last updated by author(s): Dec 12, 2023

Reporting Summary

Nature Portfolio wishes to improve the reproducibility of the work that we publish. This form provides structure for consistency and transparency in reporting. For further information on Nature Portfolio policies, see our Editorial Policies and the Editorial Policy Checklist.

Statistics

For all statistical analyses, confirm that the following items are present in the figure legend, table legend, main text, or Methods section.

n/a	Confirmed	
☐	☒	The exact sample size (*n*) for each experimental group/condition, given as a discrete number and unit of measurement
☐	☒	A statement on whether measurements were taken from distinct samples or whether the same sample was measured repeatedly
☐	☒	The statistical test(s) used AND whether they are one- or two-sided<br>*Only common tests should be described solely by name; describe more complex techniques in the Methods section.*
☐	☒	A description of all covariates tested
☐	☒	A description of any assumptions or corrections, such as tests of normality and adjustment for multiple comparisons
☐	☒	A full description of the statistical parameters including central tendency (e.g. means) or other basic estimates (e.g. regression coefficient) AND variation (e.g. standard deviation) or associated estimates of uncertainty (e.g. confidence intervals)
☐	☒	For null hypothesis testing, the test statistic (e.g. *F*, *t*, *r*) with confidence intervals, effect sizes, degrees of freedom and *P* value noted<br>*Give P values as exact values whenever suitable.*
☒	☐	For Bayesian analysis, information on the choice of priors and Markov chain Monte Carlo settings
☒	☐	For hierarchical and complex designs, identification of the appropriate level for tests and full reporting of outcomes
☒	☐	Estimates of effect sizes (e.g. Cohen's *d*, Pearson's *r*), indicating how they were calculated

Our web collection on statistics for biologists contains articles on many of the points above.

Software and code

Policy information about availability of computer code

Data collection	No special source of data collection software was used.
Data analysis	GraphPad Prism 7.0 and Image J software were used to analyze experimental data; Kmplot was used to analyze survival of cancer patients with different levels of IDH2 expression in public database; Survexpress was used to analyze in public database for IDH2 expression in breast cancer tissues with different pathological types and clinical stages. Public databases used in this study include cBioprotal (TCGA), Oncomine, and Cancer Cell Line Encyclopedia (CCLE).

For manuscripts utilizing custom algorithms or software that are central to the research but not yet described in published literature, software must be made available to editors and reviewers. We strongly encourage code deposition in a community repository (e.g. GitHub). See the Nature Portfolio guidelines for submitting code & software for further information.

Data

Policy information about availability of data

All manuscripts must include a data availability statement. This statement should provide the following information, where applicable:

- Accession codes, unique identifiers, or web links for publicly available datasets
- A description of any restrictions on data availability
- For clinical datasets or third party data, please ensure that the statement adheres to our policy

Source file and key resource tables are provided. Datasets available in the public databases including cBioprotal (TCGA) and Oncomine were used to analyze the

potential relationship between genomic changes and mRNA expression in cancers. Kmplot was used to compare the survival of cancer patients with different levels of IDH2 expression. Survexpress was utilized to analyze IDH2 mRNA expression in breast cancer tissues with different pathological types and clinical stages. The Cancer Cell Line Encyclopedia (CCLE) was used to analyze the correlation between gene copy numbers and mRNA expression.

Research involving human participants, their data, or biological material

Policy information about studies with human participants or human data. See also policy information about sex, gender (identity/presentation), and sexual orientation and race, ethnicity and racism.

Reporting on sex and gender	This study did not involve human participants or collection of human data.
Reporting on race, ethnicity, or other socially relevant groupings	This study did not involve human participants or collection of human data.
Population characteristics	This study did not involve human participants or collection of human data.
Recruitment	This study did not involve human participants or collection of human data.
Ethics oversight	This study did not involve human participants or collection of human data.

Note that full information on the approval of the study protocol must also be provided in the manuscript.

Field-specific reporting

Please select the one below that is the best fit for your research. If you are not sure, read the appropriate sections before making your selection.

☒ Life sciences ☐ Behavioural & social sciences ☐ Ecological, evolutionary & environmental sciences

For a reference copy of the document with all sections, see nature.com/documents/nr-reporting-summary-flat.pdf

Life sciences study design

All studies must disclose on these points even when the disclosure is negative.

Sample size	No statistical methods were used to predetermine sample size. The numbers of mice used in this study were chosen based on our prior experience using similar animal models. We also considered the E value between 10 and 20 as a guideline to estimate the number of animals, according to the publication by Charan & Kantharia (How to calculate sample size in animal studies? PMID: 24250214)
Data exclusions	None
Replication	Most experiments were replicated at least twice (n=3); the metabolic flux analysis using [C-13]glutamine tracing and western blot analyses of protein expression were repeated once independently on different days.
Randomization	In this study, samples were randomly allocated into experimental groups; for animal study, mice of similar age and body weights ware allocated to the control and testing groups at random.
Blinding	The investigators were not blinded to the group allocation during data collection and analysis. Blinding was not possible in this study since the same investigators performed the experiments and data analysis.

Reporting for specific materials, systems and methods

We require information from authors about some types of materials, experimental systems and methods used in many studies. Here, indicate whether each material, system or method listed is relevant to your study. If you are not sure if a list item applies to your research, read the appropriate section before selecting a response.

Materials & experimental systems

n/a	Involved in the study	
☐	☒	Antibodies
☐	☒	Eukaryotic cell lines
☒	☐	Palaeontology and archaeology
☐	☒	Animals and other organisms
☒	☐	Clinical data
☒	☐	Dual use research of concern
☒	☐	Plants

Methods

n/a	Involved in the study	
☒	☐	ChIP-seq
☐	☒	Flow cytometry
☒	☐	MRI-based neuroimaging

Antibodies

Antibodies used

IDH2 Abcam ab55271, RRID:AB_943793
LDHA Abcam ab47010, RRID:AB_1952042
HIF1 Abcam ab51608,RRID:AB_880418
ALDOA Abcam ab169544, RRID:NA
β-Actin Abcam ab6276, RRID:AB_2223210
MCL1 Abcam ab32087, RRID:AB_776245
BCL2 Abcam ab32124 ,RRID:AB_725644
β-Catenin Cell Signaling Technology CST8480,RRID:AB_11127855
Snail Cell Signaling Technology CST3879,RRID:AB_2255011
E-Cadherin Cell Signaling Technology CST3195,RRID:AB_2291471
N-Cadherin Cell Signaling Technology CST13116,RRID:AB_2687616
all were used at 1:1000

Validation

IDH2 antibody validated by knockout study
LDHA antibody ab47010 cross-reacts with LDHA, B and C.
HIF1 antibody ab51608 recognizes HIF-1-alpha
β-actin antibody validated by knockout
MCL1 antibody validated by knockout
BCL2 antibody validated by knockout
Catenin antibody used in PMID: 37795382
Snail antibody used in PMID: 37594118
E-cadherin antibody used in PMID: 37795382
N-cadherin antibody used in PMID: 37664655

Eukaryotic cell lines

Policy information about cell lines and Sex and Gender in Research

Cell line source(s)

MDA-MB-231, ATCC RRID:CVCL_0062
HCC38, ATCC RRID:CVCL_1267
BT549, ATCC RRID:CVCL_1092
MCF10A, ATCC RRID:CVCL_0598
MDA-MB-231, ATCC RRID:CVCL_0062
BT474 and MCF7 cell lines, from Chinese National Infrastructure of Cell Line Resource

Authentication

Authentication by the cell bank using STR method

Mycoplasma contamination

Routine Mycoplasma test of our lab, negative.

Commonly misidentified lines
(See ICLAC register)

None

Animals and other research organisms

Policy information about studies involving animals; ARRIVE guidelines recommended for reporting animal research, and Sex and Gender in Research

Laboratory animals

Mice: Balb/C-nude, female, 6-8 week; Animal housing conditions: dark/light cycle = 12h/12h; ambient temperature 2-26?; humidity 40-70%.

Wild animals

None

Reporting on sex

This study focused on triple-negative breast cancer (TNBC). Since TNBC occurs mostly in woman, we used female mice in our study.

Field-collected samples

None

Ethics oversight	The animal study protocol for this research was approved by the Institutional Animal Care and Research Ethics Committee, Sun Yat-sen University Cancer Center (Guangzhou, China).

Note that full information on the approval of the study protocol must also be provided in the manuscript.

Plants

Seed stocks	No plants were used in this study.
Novel plant genotypes	No plants were used in this study.
Authentication	No plants were used in this study.

Flow Cytometry

Plots

Confirm that:

☒ The axis labels state the marker and fluorochrome used (e.g. CD4-FITC).

☒ The axis scales are clearly visible. Include numbers along axes only for bottom left plot of group (a 'group' is an analysis of identical markers).

☒ All plots are contour plots with outliers or pseudocolor plots.

☒ A numerical value for number of cells or percentage (with statistics) is provided.

Methodology

Sample preparation	Cells were harvested and washed with PBS twice.
Instrument	Beckman cytoFLEX flow cytometer (BD Biosciences)
Software	Beckman cytoFLEX flow cytometer (BD Biosciences)
Cell population abundance	All cells were subjected to flow cytometer analysis
Gating strategy	All cells were subjected to flow cytometer analysis

☒ Tick this box to confirm that a figure exemplifying the gating strategy is provided in the Supplementary Information.
